# Screening of Pleural Mesothelioma Cell Lines for Kinase Activity May Identify New Mechanisms of Therapy Resistance in Patients Receiving Platin-Based Chemotherapy

**DOI:** 10.1155/2019/2902985

**Published:** 2019-12-23

**Authors:** Sabrina Borchert, Pia-Maria Suckrau, Michael Wessolly, Elena Mairinger, Balazs Hegedus, Thomas Hager, Thomas Herold, Wildfried E. E. Eberhardt, Jeremias Wohlschlaeger, Clemens Aigner, Agnes Bankfalvi, Kurt Werner Schmid, Robert F. H. Walter, Fabian D. Mairinger

**Affiliations:** ^1^Institute of Pathology, University Hospital Essen, University of Duisburg-Essen, Essen, Germany; ^2^German Cancer Consortium (DKTK), Partner Site University Hospital Essen, Hufelandstrasse 55, 45122 Essen, Germany; ^3^Department of Thoracic Surgery and Thoracical Endoscopy, Ruhrlandklinik, West German Lung Centre, University Hospital Essen, University of Duisburg-Essen, Essen, Germany; ^4^Department of Medical Oncology, West German Cancer Center, University Hospital Essen, University of Duisburg-Essen, Essen, Germany; ^5^Ruhrlandklinik, West German Lung Centre, University Hospital Essen, University of Duisburg-Essen, Essen, Germany; ^6^Department of Pathology, Diakonissenkrankenhaus Flensburg, Flensburg, Germany

## Abstract

**Background:**

Malignant pleural mesothelioma (MPM) is a rare, predominantly asbestos-related and biologically highly aggressive tumor associated with a dismal prognosis. Multimodal therapy consisting of platinum-based chemotherapy is the treatment of choice. The reasons underlying the rather poor efficacy of platinum compounds remain largely unknown. Kinase activity might influence cellular response to these regimens.

**Materials and Methods:**

For this exploratory study, we screened MPM cell lines (NCI-H2452, NCI-H2052, and MSTO-211H) differing in response to cisplatin and benign control fibroblasts (MRC-5) for overall phosphorylation patterns as well as kinase activity with respect to cellular response to cisplatin-based therapeutics. We analysed the cell lines for cellular kinases in a high-throughput manner using the highly innovative technique PamGene. Cell state analysis including apoptosis, necrosis, and cell viability was performed by using enzyme activity and fluorescent-based assays.

**Results:**

Cisplatin alters cellular phosphorylation patterns affecting cell cycle, migration, adhesion, signal transduction, immune modulation, and apoptosis. In cisplatin-responsive cell lines, phosphorylation of AKT1 and GSK3B was decreased but could not be influenced in cisplatin-resistant NCI-H2452 cells. Cisplatin-responsive cell lines showed increased phosphorylation levels of JNK1/2/3 but decreased phosphorylation in cisplatin-resistant NCI-H2452 cells.

**Conclusion:**

Kinase phosphorylation and activity might play a crucial role in cellular response to cytostatic agents. Cisplatin influences phosphorylation patterns with distinct features in cisplatin-resistant cells. These alterations exert a significant impact on cell cycle, migration, adhesion, signal transduction, immune modulation, and apoptosis of the respective tumor cells. Based on our results, the induction of p38 or JNK1/3, or inhibition of AKT1 by, for example, BIA-6, might offer a positive synergistic effect by induction of an apoptotic response to cisplatin-based treatment, thus potentially enhancing the clinical outcome of MPM patients.

## 1. Introduction

Malignant pleural mesothelioma (MPM) is a rare, predominantly asbestos-related tumor and associated with a dismal prognosis [1, 2]. In the US, approximately 2500 new cases of mesothelioma are diagnosed each year and the incidence of mesothelioma is expected to decline steadily [1, 3, 4]. In contrast, the incidence of mesothelioma in Europe continues to rise [1, 2, 5–7].

Besides pemetrexed, platinum compounds are standard chemotherapeutic agents and still a hallmark of chemotherapy for MPM [8]. In clinical practice, pemetrexed is used in combination with cisplatin [9] or carboplatin [10–13]. Platinum-containing regimens have a greater activity than nonplatinum containing combinations [14]. Cisplatin treatment shows a response rate of merely 14% and a median survival of fewer than 7 months [15]. Carboplatin treatment results in similar response rates ranging from 6 to 16% [15]. The reasons for this rather poor efficacy of platinum compounds are incompletely understood until now.

Platinum cytotoxicity is based on forming bulky DNA adducts by chemically altering DNA bases by covalent binding of platinum [13], leading to both DNA interstrand and (1 and 2 or 1 and 3) intrastrand cross-linking [16–23]. Platinum compounds prevent normal cell replication and trigger apoptosis [18, 22, 24], unless adducts from genomic DNA are repaired [21].

Resistance to antitumor agents such as platin compounds has been correlated to a broad spectrum of mechanisms. It is known since the early 1990s that the activity of several proteins involved in the development of antitumor drug resistance is regulated by protein phosphorylation [25]. Especially, the role of protein kinase C and others has been previously described [26]. During the past decade, our understanding of the underlying effect of platin-induced apoptosis has increased greatly by the identification of some of the major components involved in apoptosis and the processes regulating their activation. Kinases that have been suggested to play a role in apoptosis encompass the mitogen-activated protein kinase (MAPK) family, specifically p42/44 ERK, p38 MAPK, c-Jun N-terminal kinase (JNK), cyclic AMP-dependent protein kinase (PKA), protein kinase B (PKB), or AKT and protein kinase C (PKC) [27]. Furthermore, phosphorylation levels of different DNA damage genes such as ATM or ATR are known to influence cellular response to replicative stress induced by platinum containing drugs [28]. In addition, it has been shown in ovarian cancer and sarcoma cells expressing constitutively active JAK2 that cisplatin significantly inhibited tyrosine phosphorylation and kinase activity of JAK2 in a dose- and time-dependent manner [29].

Against this background, we aimed to investigate the impact of overall phosphorylation patterns as well as kinase activity in cellular response to cisplatin-based therapeutics. Therefore, we analysed different MPM cell lines for cellular kinases in a high-throughput manner using the highly innovative technique PamGene.

## 2. Materials and Methods

### 2.1. Cell Culture

MPM cell lines MSTO-211H (biphasic subtype and moderately cisplatin sensitive) and NCI-H2052 (epithelioid subtype and cisplatin sensitive) as well as the cell line NCI-H2452 (sarcomatoid subtype and cisplatin-resistant) were cultured in Roswell Park Memorial Institute (RPMI)-1640 medium (Thermo Fisher Scientific, Massachusetts, USA). The human lung-fibroblast cell line MRC-5 was used as a control cell line. MRC-5 cells were cultured in Minimal Essential Medium (Thermo Fisher Scientific). All culture media were supplemented with 10% fetal calf serum and 1% penicillin and streptomycin (Thermo Fisher Scientific).

### 2.2. Treatment of MPM Cell Lines with Cisplatin

The effect of cisplatin on kinase activity was analysed in each cell line. Therefore, 1.6 × 10^5^ cells/well were seeded in a 24-well plate. After 12 h of incubation at 37°C and 5% CO_2_, 10 *μ*M of cisplatin (Selleckchem, Houston, USA) was added to cells. After 48 h of incubation with cisplatin, protein isolation was performed according to the protocol 1160 from the PamGene platform (PamGene International B. V., Wolvenhoek, Netherlands). Therefore, cells were lysed by using M-PER Mammalian Protein Extraction Reagent containing HALT phosphatase inhibitor cocktail and HALT protease inhibitor cocktail EDTA-free (Thermo Fisher Scientific). Lysed cells were harvested by using a cell scraper. Lysates were stored in 5–20 *μ*l aliquots at −80°C.

The protein concentration was determined via fluorometric quantification (Qubit, Thermo Fisher Scientific) using the protein assay kit according to the manufacturers' instructions.

### 2.3. Protein Tyrosine Kinase Assay

The Protein Tyrosine Kinase Assay (PTK Assay, PamGene) was performed according to the manufacturers' instructions. The PamChip®-4 was prepared by a blocking step with 30 *μ*l of 2% BSA (PamGene). Master mix was prepared by using the reagent kit for PTK PamChip arrays (PamGene). 5 *μ*g of sample protein lysate was applied. As required for the mastermix, ATP (4 mM) was diluted 1 : 25.

### 2.4. Serine/Threonine Kinase Assay

The Serine/Threonine Kinase Assay (STK Assay, PamGene) was performed according to the manufacturers' instructions. The PamChip®-4 was prepared by a blocking step with 30 *μ*l of 2% BSA (PamGene). Master mix was prepared by using the reagent kit for STK PamChip arrays (PamGene). 0.5 *μ*g of sample protein lysate was applied. As required for the mastermix, ATP (4 mM) was diluted 1 : 25.

### 2.5. Kinase Activity Determination

Analysis of the results of PTK and STK assay was performed by using the BioNavigator software (PamGene).

Image analysis and log2 transformation of the results were performed by using the BioNavigator software (PamGene). Based on substrate phosphorylation pattern, kinase activities of each specific kinase were estimated using the kinase upstream analysis algorithm (BioNavigator). Each corresponding kinase was classified by specificity of each kinase, and dependency power levels were calculated. To visualize kinase activity changes before and after cisplatin treatment, kinase trees were generated by using the KinHub platform (http://www.kinhub.org/kinmap/).

### 2.6. Statistical Analysis

Statistical and graphical analyses of specific phosphosite phosphorylation levels were performed with the *R* statistical programming environment (v3.2.3).

Before starting the explorative data analysis, the Shapiro–Wilk test was applied to test for normal distribution of the data. Based on the results, either parametric or nonparametric test was used. For dichotomous variables, either the Wilcoxon Mann–Whitney rank sum test (nonparametric) or two-sided student's *t*-test (parametric) was applied. For ordinal variables with more than two groups, either the Kruskal–Wallis test (nonparametric) or ANOVA (parametric) was used to detect group differences.

Double dichotomous contingency tables were analysed using Fisher's exact test. To test dependency of ranked parameters with more than two groups, Pearson's chi-squared test was used. Correlations between metric variables were tested by using Spearman's rank correlation test as well as Pearson's product moment correlation coefficient for linear modelling.

Due to the multiple statistical testing, all *p* values were adjusted by using the false discovery rate (FDR). The level of statistical significance was defined as *p* ≤ 0.05 after adjustment.

## 3. Results

### 3.1. Cisplatin Treatment Reveals Differences in Phosphorylation Pattern

**Figure 1:**
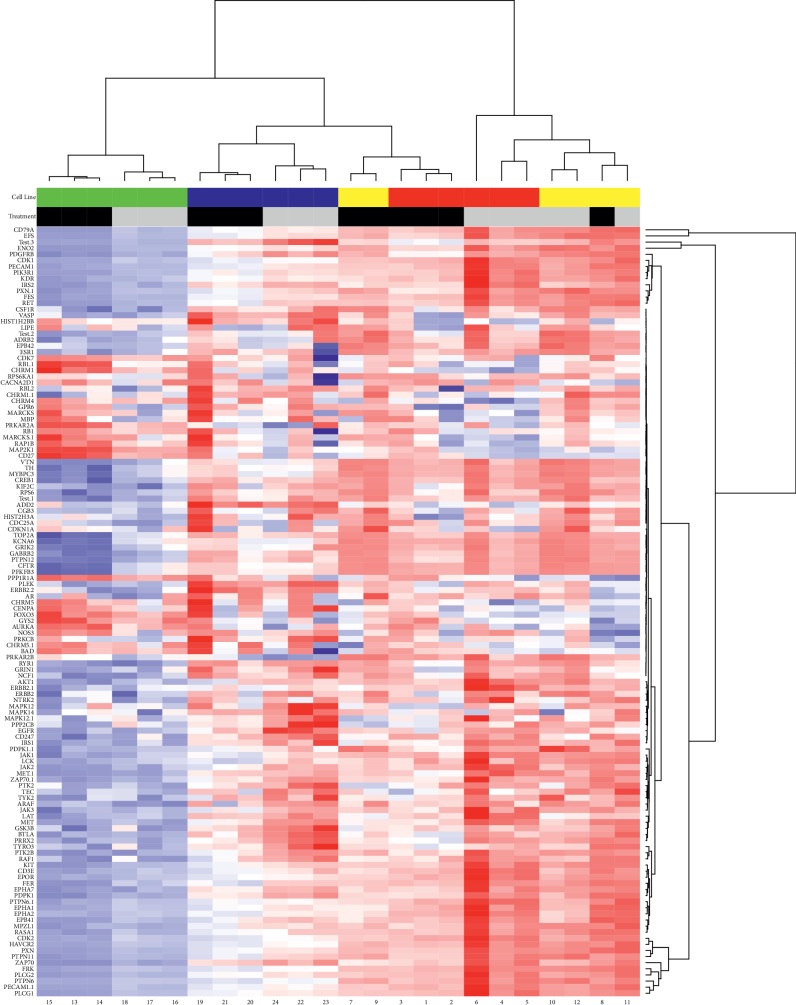
Heatmap of phosphorylation pattern between cells and treatment. MRC-5 is depicted by the green indicator on the *y*-axis and shows minor phosphorylation changes when comparing cisplatin treatment and medium. Blue indicators (*y*-axis) depict MSTO-211H and show that this cell line presented with changes in phosphorylation during cisplatin therapy. MSTO-211H cells have a distinct phosphorylation pattern compared to the other MPM cell lines. During therapy, a slight shift towards the phosphorylation pattern of the other two MPM cell lines can be seen. NCI-H2052 (shown in red) and NCI-H2452 (shown in yellow) present with an overlap in their phosphorylation pattern and show a much stronger phosphorylation of multiple phosphosites during therapy. Contrarily, MSTO-211H cells showed intermediate to slightly elevated phosphorylation. In contrast, MRC-5 cells present in general with minor phosphorylation regardless of the respective phosphosite and treatment.

MRC-5 cells showed minor changes in phosphorylation of phosphosites when comparing cisplatin treatment (highlighted by black bars) and medium (highlighted by grey bars) (Figure 1, green bars). MSTO-211H cells (blue bars) presented with changes in phosphorylation during cisplatin therapy. MSTO-211H has a distinct cluster in its phosphorylation pattern compared to the other MPM cell lines. During therapy, a similar shift in the phosphorylation pattern could be observed compared to the other two MPM cell lines. Untreated, a similar phosphorylation pattern could be detected for MSTO-211H cells. However, certain phosphosites showed a significantly enhanced signal. NCI-H2052 (red bars) and NCI-H2452 (yellow bars) presented with an overlap in their phosphorylation pattern and showed a much stronger phosphorylation of multiple phosphosites without exposition to cisplatin. MRC-5 presented in general with minor phosphorylation regardless of the respective phosphosites and treatment.

**Figure 2:**
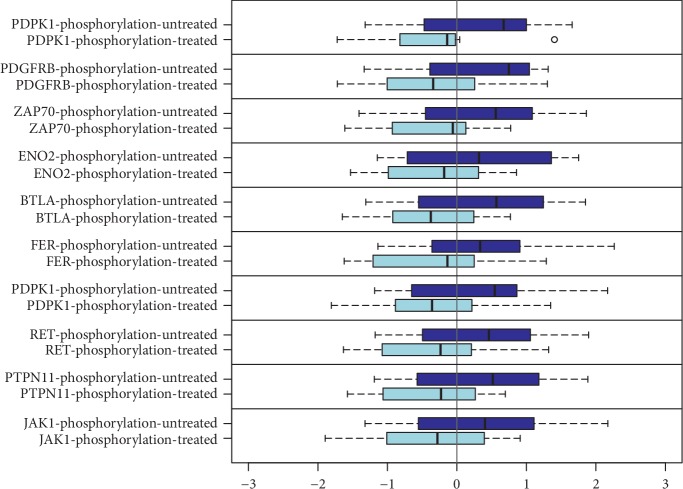
Top 10 influenced phosphosites during cisplatin therapy. On the *y*-axis, phosphosites of the respective protein are shown. Each box summarizes the results for all four cell lines, being measured in triplicates (dark blue = medium and light blue = cisplatin). To make the results comparable, the *x*-axis depicts a dimensionless *Z*-score. A significant, global reduction of tyrosine phosphosite phosphorylation could be observed. No significance for serine/threonine kinases was monitored. The figure focusses on the top 10 changes because further boxplots would be repetitive. In sum, 77 phosphosites showed significantly altered phosphorylation during cisplatin therapy. *p* values are depicted in Suppl. [Supplementary-material supplementary-material-1].

In sum, 54 phosphosites showed significantly altered phosphorylation during cisplatin therapy. 52 showed a reduction in phosphorylation status due to treatment, whereas two (PPR1A and FOXO3) showed an induction in their phosphorylation. In Figure 2, ten phosphosites with the most significant changes in phosphorylation after treatment with cisplatin are shown (*p* values are shown in Suppl. [Supplementary-material supplementary-material-1]). A significant global reduction of tyrosine phosphosite phosphorylation could be outlined. No biologically relevant significance for serine/threonine kinases was monitored.

The comparison of significantly altered phosphorylation levels between the different cell lines revealed 62 alterations. The majority of differences [29] could be observed between MSTO-211H and the MRC-5 control cell line (Suppl. [Supplementary-material supplementary-material-1]). In line with the results visualized in the heatmap, all targets showed significantly reduced signals. Between NCI-H2452 and MRC-5, 15 differences were observed, again, all with lower phosphorylation levels. Also, between NCI-H2052 and MRC-5, ten significantly higher phosphorylated sites could be observed in the tumor cells. Between MSTO-211H and NCI-H2452, three phosphosites (CD3Z, EGFR, and GSK3ß) showed a higher phosphorylation and four phosphosites (EFS, ENOG, EPHA7, and PTN6) showed lower phosphorylation levels in NCI-H2452 cells. Between NCI-H2052 vs. MSTO-211H, as well as between NCI-H2052 vs. NCI-H2452, no significantly altered phosphorylation levels could be observed.

### 3.2. Influence of Different Phosphorylation Patterns in Response to Cisplatin

**Figure 3:**
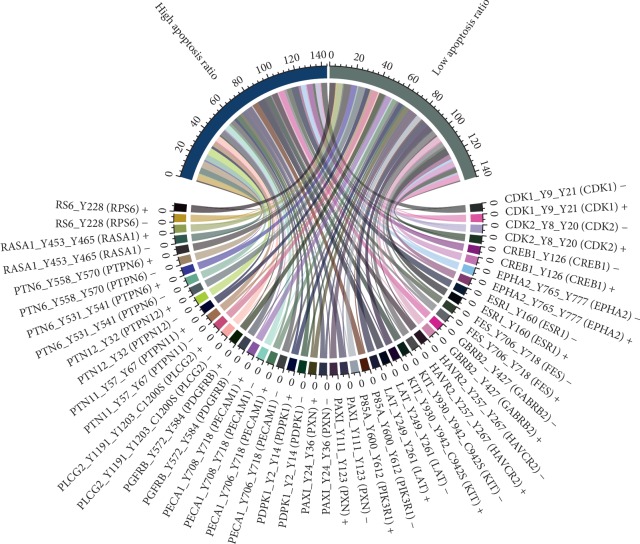
Circos plot of high and low phosphorylated phosphosites associated with high or low apoptosis ratio after cisplatin treatment. High and low phosphorylation of phosphosites were indicated with “+” or “−”.

Overall, high phosphorylation of phosphosites (Suppl. [Supplementary-material supplementary-material-1]) lead to resistance against cisplatin therapy. In general, 24 phosphosites seem to impact cellular response to cisplatin-based therapeutic regimens. Respective phosphosites are shown in Suppl. [Supplementary-material supplementary-material-1]. Especially, high phosphorylation of ESR1, LAT, PTN12, and PTN6 showed the strongest apoptosis-preventing effect. The circos plot (Figure 3) depicts the frequency of high or low phosphorylated phosphosites of kinases and their responsiveness to cisplatin.

### 3.3. Biological Relevance and Effected Cellular Pathways

Analysis of the phosphorylation pattern with respect to MAPK signaling pathway (KEGG hsa04010), cell cycle (KEGG hsa04110), and pathways in cancer (KEGG hsa05200) was performed for each cell line. Induction of phosphorylation by cisplatin is indicated by green labels, and reduction of phosphorylation is indicated in red labels (Suppl. Figures [Supplementary-material supplementary-material-1]–[Supplementary-material supplementary-material-1]). In the MAPK signaling pathway, cisplatin reduces phosphorylation of receptor tyrosine kinases (especially EGFR, EPHA2, and KDR), and RASA1, RAF1, and AKT1 in each cell line. In the cell cycle pathway, GSK3B, CDK2, and CDK1 are reduced by cisplatin in each cell line, and in cancer pathways, cisplatin reduces phosphorylation of GSK3B, AKT1, PDGFRa/b, PLCG1/2, and CDK2. In NCI-H2452 cells, the reduction of phosphorylation by cisplatin is weaker than in other cell lines (indicated by light red).

Proteins contributing to cell adhesion/migration and membrane properties showed a cluster with remarkable response to cisplatin treatment in NCI-H2052 and MSTO-211H. EPHA1 (Suppl. [Supplementary-material supplementary-material-1]), EPHA2 (Suppl. [Supplementary-material supplementary-material-1]), EPHA7 (Suppl. [Supplementary-material supplementary-material-1]), EFS (Suppl. [Supplementary-material supplementary-material-1]), EPB41, PTK2B (Suppl. [Supplementary-material supplementary-material-1]), FER, FES, KIT (Suppl. [Supplementary-material supplementary-material-1]), PXN, PECAM1, PDGFRB, KDR (Suppl. [Supplementary-material supplementary-material-1]), and ZAP70 showed comparable phosphorylation changes during cisplatin therapy (all *p* < 0.01 after Bonferroni correction; Table 1). Phosphorylation of the PTK phosphosites was low in fibroblasts in general. In NCI-H2452, cisplatin therapy led to a low reduction of phosphorylation with respect to the mentioned proteins. Instead, NCI-H2052 showed a much stronger reduction of phosphorylation of the mentioned phosphosites. MSTO-211H showed an intermediate to strong response during cisplatin therapy with respect to reduction of phosphorylation. Cisplatin therapy led to major changes in EFS phosphorylation, as phosphorylation decreased in all cell lines in a remarkable manner (*p* < 0.0001). In MRC-5 cells, the phosphosite was not anymore phosphorylated during cisplatin treatment. The result for EFS is shown in Suppl. [Supplementary-material supplementary-material-1].

Cisplatin leads to a reduction of AKT1 phosphorylation influencing one of the three AKT1 phosphosites (Suppl. [Supplementary-material supplementary-material-1]). Phosphorylation was low in MRC-5 cells, regardless of the treatment. NCI-H2052 showed the strongest changes in AKT1 phosphorylation during therapy. Similar results were found for PTK2B (Suppl. [Supplementary-material supplementary-material-1]), which is an upstream regulator of AKT1. Phosphorylation reduced during cisplatin therapy in NCI-H2052 and MSTO-211H, whereas the other cell lines showed minor response. PDGFRB and KDR phosphorylation showed comparable phosphorylation changes as mentioned above. Both contribute to the activation of AKT1.

Additional proteins that mediate inter- and intracellular signal transduction (ARAF, EPHA1, EPHA2, EPHA7, KIT, PTPN11, PIK3R1, PTPN6, and KDR) showed similar results as depicted in Suppl. Figures [Supplementary-material supplementary-material-1]–[Supplementary-material supplementary-material-1]. The changes in phosphorylation are redundant to the results mentioned before.

BTLA, CD3E, CD247, CD79A, PTK2B, HAVCR2, PECAM1, and ZAP70 modulate immune response and showed differential phosphorylation during therapy. NCI-H2052 showed the strongest decrease in phosphorylation of these proteins during cisplatin therapy. In addition, phosphorylation was much higher before therapy compared to the other cell lines investigated. Similar high phosphorylation was also found in NCI-H2452 before therapy, but phosphorylation reduced less during therapy compared to NCI-H2052. Phosphorylation in MSTO-211H was much lower compared to the other two MPM cell lines. During therapy, only a minor reduction of phosphorylation was monitored for proteins modulating immune response. MRC-5 phosphorylation was low in general but decreased during therapy to a nonmeasurable extent.

Phosphorylation of proteins driving cell cycle control showed major changes during therapy (CDK1, CDK2, EPHA1, EPHA2, EPHA7, ENO2, PTK2B, FER, FES, FRK, KIT, PDGFRB, and KDR). The changes were similar to the above presented results. Again, NCI-H2052 showed the strongest changes, followed by NCI-H2452. Similar, but with general lower phosphorylation, MSTO-211H was comparable with the other two MPM cell lines. Again, MRC-5 presented with generally low phosphorylation of the reported proteins and reduced during therapy—in most cases, phosphorylation was absent due to cisplatin treatment.

### 3.4. Upstream Kinase Analysis

Kinase trees were created for each cell line (Suppl. Figures [Supplementary-material supplementary-material-1]–[Supplementary-material supplementary-material-1]). The most affected kinases were in the family of tyrosine kinases (e.g., ALK, FES, and ZAP70) and CMGC kinases (e.g., CDK1, CDK2, and ERK1) in all four cell lines.

As depicted in the score plots and volcano plots (Suppl. Figures [Supplementary-material supplementary-material-1]–[Supplementary-material supplementary-material-1]), NCI-H2052 cells showed a 2.5–3-fold decrease in kinase activity of FGFR1, FES, and ALK due to cisplatin treatment, with a high specificity score (2, dark red) for the respective phosphosites. In NCI-H2052, as well as in MRC-5 cells, kinase activity of ERK1/2 and CDK1 was 2.3-fold increased due to cisplatin treatment. In MRC-5 cells, kinase activity of HER2, FLT3, and EGFR showed a very strong decrease (6–9.5-fold) with a high specificity (2, dark red) for ten respective phosphosites. In MSTO-211H cells, kinase activity was decreased (3-4-fold) by 10 phosphosites with high specificity. Kinase activity was slightly decreased (0.4–0.7-fold) in NCI-H2452 for FAK1, Ron, SRC, CK1, and COT.

## 4. Discussion

Platinum compounds are standard chemotherapeutic agents and still a hallmark of chemotherapy for MPM in combination with pemetrexed [30]. Nevertheless, platin-containing regimens show unsatisfying response. Therefore, we investigated MPM cell lines, differing in their response to cisplatin (NCI-H2052: high apoptotic response, MSTO-211H: sparsely apoptotic response, and NCI-H2452: no response). We screened the cells for overall phosphorylation patterns as well as kinase activity with respect to cellular response to cisplatin-based therapeutics. We analysed the cell lines for cellular kinases in a high-throughput manner using the highly innovative technique PamGene.

In our study, we could demonstrate differences in the phosphorylation pattern in all cell lines due to cisplatin treatment. Overall, increase in phosphorylation after addition of cisplatin indicate an adaptive mechanism to escape from the effect of cisplatin. In particular, high phosphorylation of ESR1, LAT, PTN12, and PTN6 showed antiapoptotic effects. PTN12 has dephosphorylation functions and therefore influences cellular signaling cascades [31]. It dephosphorylates cellular tyrosine kinases like ERBB2 and PTK2B. ERBB2 encodes HER2/neu that inhibits apoptosis by stimulation of proliferation via the RAS-MAP kinase pathway [32, 33]. In NCI-H2452 cells, phosphorylation levels of ERBB2 is not reduced, compared to other cell lines. Therefore, it could be suggested that this mechanism plays a role in this cell line, supporting its cisplatin resistance.

BTLA, CD3E, CD247, CD79A, PTK2B, HAVCR2, PECAM1, and ZAP70 modulate immune response and showed differential phosphorylation during therapy. The activation of BTLA leads to inhibition of CD8^+^ cancer-specific T-cells [34]. BTLA showed decreased phosphorylation levels in all cell lines, but we could not detect any cellular effects of the differential phosphorylation patterns in cell state analysis. Nevertheless, we hypothesize this to be an important factor in MPM patients regarding cisplatin treatment. Resistance mechanisms possibly be challenged by kinase inhibitors, regulating immune response to cisplatin.

Gao et al. found that elevated expression and phosphorylation of AKT by GSK3B and PTEN was correlated with cell viability, migration, and apoptosis, and this might be explained by chemoresistance in breast cancer [35]. In NCI-H2052, we could reduce phosphorylation of AKT1 and GSK3B by cisplatin and therefore could induce apoptosis in this cell line. Cisplatin-treated and untreated NCI-H2452 cells showed no significant changes between phosphorylation of AKT1. Benzothienopyrimidine derivative (BIA-6), an AKT inhibitor, could effectively block the PI3K/AKT pathway in lung cancer cells in a dose-dependent manner and thus increase apoptosis [36]. Based on our data, a possible synergistic effect with platin-based treatment can be suggested. It could be possible that BIA-6 might also improve efficiency of cisplatin in NCI-H2452 cells.

In NCI-H2052, as well as in MRC-5 cells, kinase activity of p38 and ERK1/2 was increased due to cisplatin treatment. Hsieh et al. also assessed increased phosphorylation of ERK1/2 and p38 in nasopharyngeal carcinoma (NPC) and observed this effect by activation of caspases [37]. This confirms to our observation since NCI-H2052 and MRC-5 showed the highest apoptotic response to cisplatin. MSTO-211H, with lower apoptosis rates, showed only a slight increase of activity of p38 and ERK1/2, whereas nonresponding NCI-H2452 cells showed decreased p38 kinase activity.

Zhao et al. observed associations between increased apoptosis by high expression of phosphorylated c-Jun N-terminal kinase (JNK) and subsequently elevated expression levels of p53 in ovarian cancer cells during treatment of platinum containing drugs [38]. This supports our observation in NCI-H2052 and MRC-5, showing elevated activity of JNK1/2/3 and decreased phosphorylation of JNK1/3 in cisplatin-resistant NCI-H2452 cells. Bar et al. found the activating transcription factor 3 (ATF3) as an important regulator of cisplatin cytotoxicity, being activated in platin-sensitive lung cancer cells due to cisplatin treatment [39]. In platin-sensitive cells, cisplatin induced activation of JNK and thus ATF3 induction. In their tested resistant cell lines, this JNK induction was missed. In their study, they tested the FDA-approved histone deacetylase inhibitor vorinostat demonstrating synergistic cytotoxicity in lung cancer cell lines Calu-6 and NCI-H23 cells together with cisplatin. As NCI-H2452 cells also show still activity of JNK, it would be interesting to test this histone deacetylase inhibitor also in this cell line.

## 5. Conclusions

Kinase phosphorylation and activity might play a crucial role in cellular response to cytostatic agents. Cisplatin treatment results in altered phosphorylation patterns in both the MPM cell lines and the lung fibroblast cell line. These alterations have consequences for cell cycle, migration, adhesion, signal transduction, immune modulation, and apoptosis of the cell. Cisplatin-resistant MPM cells showed a clearly distinct phosphorylation pattern compared to cells showing response to cisplatin, indicating a specific sensitivity-profile. Our results indicate that inhibition of AKT1 by, e.g., BIA-6, or, in another approach, induction of p38 or JNK1/3 of the MAPK pathway, might offer positive synergistic effects through induction of an apoptotic response to cisplatin-based treatment and thus potentially enhance patients' clinical outcome.

## Figures and Tables

**Table 1:** Significant phosphorylation changes after cisplatin treatment (*p* values and Bonferroni-adjusted *p* values).

Phosphosites	*p* value	Bonferroni-adj. *p* value
Tyrosine-protein phosphatase nonreceptor type 11 (EC 3.1.3.48) (protein-tyrosine phosphatase 2C) (PTP-2C) (PTP-1D) (SH-PTP3) (SH-PTP2) (SHP-2) (Shp2)._PTN11_57_67_Q06124	1.58*E* − 10	1.40*E* − 08
Gamma-enolase (EC 4.2.1.11) (2-phospho-D-glycerate hydrolyase) (neural enolase) (neuron-specific enolase) (NSE) (enolase 2)_ENOG_37_49_P09104	2.23*E* − 10	1.99*E* − 08
Tyrosine-protein phosphatase nonreceptor type 6 (EC:3.1.3.48)_PTN6_531_541_P29350	6.41*E* − 09	5.71*E* − 07
Paxillin._PAXI_24_36_P49023	8.74*E* − 09	7.78*E* − 07
Tyrosine-protein kinase JAK2 (EC 2.7.10.2) (janus kinase 2) (JAK-2)_JAK2_563_577_O60674	3.28*E* − 08	2.92*E* − 06
Embryonal fyn-associated substrate (HEFS)_EFS_246_258_O43281	3.38*E* − 08	3.01*E* − 06
Hepatitis A virus cellular receptor 2, T-cell immunoglobulin and mucin domain-containing protein 3, T-cell membrane protein 3_HAVR2_257_267_Q8TDQ0	4.21*E* − 08	3.75*E* − 06
Proto-oncogene tyrosine-protein kinase fes/Fps (EC 2.7.10.2) (C-Fes)_FES_706_718_P07332	5.51*E* − 08	4.91*E* − 06
B-cell antigen receptor complex-associated protein alpha-chain precursor (Ig-alpha) (MB-1 membrane glycoprotein) (surface IgM-associated protein) (membrane-bound immunoglobulin-associated protein) (CD79a antigen)_CD79 A_181_193_P11912	5.72*E* − 08	5.09*E* − 06
Tyrosine-protein kinase FRK (EC 2.7.10.2) (FYN-related kinase) (nuclear tyrosine protein kinase RAK)_FRK_380_392_P42685	6.73*E* − 08	5.99*E* − 06
NA_ART_004_EAIYAAPFAKKKXC_NA	7.47*E* − 08	6.65*E* − 06
Tyrosine-protein kinase JAK1 (EC 2.7.10.2) (janus kinase 1) (JAK-1)_JAK1_ 1027_1039_P23458	8.05*E* − 08	7.17*E* − 06
Insulin receptor substrate 2_IRS2_626_638_Q9Y4H2	9.51*E* − 08	8.47*E* − 06
Hepatocyte growth factor receptor precursor (EC 2.7.10.1) (HGF receptor) (scatter factor receptor) (SF receptor) (HGF/SF receptor) (Met proto-oncogene tyrosine kinase) (c-Met)_MET_1228_1240_P08581	1.22*E* − 07	1.08*E* − 05
Paxillin._PAXI_111_123_P49023	1.42*E* − 07	1.26*E* − 05
Phosphatidylinositol 3-kinase regulatory subunit alpha (PI3-kinase p85 subunit alpha) (PtdIns-3-kinase p85-alpha) (PI3K)_P85 A_600_612_P27986	1.81*E* − 07	1.61*E* − 05
Proto-oncogene tyrosine-protein kinase receptor ret precursor (EC 2.7.10.1) (C-ret)_RET_1022_1034_P07949	1.89*E* − 07	1.68*E* − 05
1-Phosphatidylinositol-4,5-bisphosphate phosphodiesterase gamma-1 (EC 3.1.4.11) (phosphoinositide phospholipase C) (PLC-gamma-1) (phospholipase C-gamma-1) (PLC-II) (PLC-148)_PLCG1_764_776_P19174	2.18*E* − 07	1.94*E* − 05
Protein tyrosine kinase 2 beta (EC 2.7.10.2) (focal adhesion kinase 2) (FADK 2) (proline-rich tyrosine kinase 2) (cell adhesion kinase beta) (CAK beta) (calcium-dependent tyrosine kinase) (CADTK) (related adhesion focal tyrosine kinase) (RAFTK)_FAK2_572_584_Q14289	2.55*E* − 07	2.27*E* − 05
Linker for activation of T-cells family member 1 (36 kDa phospho-tyrosine adapter protein) (pp36) (p36-38)_LAT_249_261_O43561	2.63*E* − 07	2.34*E* − 05
Mast/stem cell growth factor receptor kit (EC:2.7.10.1), CD117_KIT_930_942_C942S_P10721	2.91*E* − 07	2.59*E* − 05
Tyrosine-protein kinase ZAP-70 (EC 2.7.10.2) (70 kDa zeta-associated protein) (syk-related tyrosine kinase)_ZAP70_313_325_P43403	2.92*E* − 07	2.60*E* − 05
Cyclin-dependent kinase 1 (EC:2.7.11.22, EC:2.7.11.23), cell division protein kinase 1, cell division control protein 2 homolog, p34 protein kinase (CDK1)_CDK1_9_21_P06493	2.94*E* − 07	2.61*E* − 05
Erythropoietin receptor precursor (EPO-R)_EPOR_361_373_P19235	4.82*E* − 07	4.29*E* − 05
1-Phosphatidylinositol 4,5-bisphosphate phosphodiesterase gamma-2 (EC:3.1.4.11) (phosphoinositide phospholipase C-gamma-2) (PLC-IV) (phospholipase C-gamma-2) (PLC-gamma-2)_PLCG2_1191_1203_C1200S_P16885	5.38*E* − 07	4.79*E* − 05
Platelet endothelial cell adhesion molecule precursor (PECAM-1) (EndoCAM) (GPIIA′) (CD31 antigen)_PECA1_708_718_P16284	5.64*E* − 07	5.02*E* − 05
Platelet endothelial cell adhesion molecule precursor (PECAM-1) (EndoCAM) (GPIIA′) (CD31 antigen)_PECA1_706_718_P16284	5.64*E* − 07	5.02*E* − 05
Cyclin-dependent kinase 2 (EC:2.7.11.22) cell division protein kinase 2 (EC 2.7.11.22) (p33 protein kinase)_CDK2_8_20_P24941	6.07*E* − 07	5.40*E* − 05
RAC-alpha serine/threonine-protein kinase (EC:2.7.11.1) (PKB, RAC)_AKT1_320_332_P31749	8.65*E* − 07	7.70*E* − 05
Ephrin type-A receptor 7 precursor (EC 2.7.10.1) (tyrosine-protein kinase receptor EHK-3) (EPH homology kinase 3) (receptor protein-tyrosine kinase HEK11)_EPHA7_607_619_Q15375	8.96*E* − 07	7.98*E* − 05
Vascular endothelial growth factor receptor 2 precursor (EC 2.7.10.1) (VEGFR-2) (kinase insert domain receptor) (protein-tyrosine kinase receptor Flk-1) (CD309 antigen)_VGFR2_989_1001_P35968	1.15*E* − 06	0.00010243
Myelin protein zero-like protein 1_MPZL1_236_246_O95297	1.16*E* − 06	0.00010287
Tyrosine-protein phosphatase nonreceptor type 6 (EC:3.1.3.48)_PTN6_558_570_P29350	1.30*E* − 06	0.00011555
Proto-oncogene tyrosine-protein kinase FER (EC 2.7.10.2) (p94-FER) (c-FER) (tyrosine kinase 3)_FER_707_719_P16591	1.47*E* − 06	0.00013057
Epidermal growth factor receptor precursor (EC 2.7.10.1) (receptor tyrosine-protein kinase ErbB-1)_EGFR_1165_1177_P00533	1.68*E* − 06	0.00014992
Paired mesoderm homeobox protein 2 (PRX-2) (paired-related homeobox protein 2)_PRRX2_202_214_Q99811	1.84*E* − 06	0.00016357
3-Phosphoinositide-dependent protein kinase 1 (EC 2.7.11.1) (hPDK1)_PDPK1_2_14_O15530	2.07*E* − 06	0.00018412
Receptor tyrosine-protein kinase erbB-2 precursor (EC 2.7.10.1) (p185erbB2) (C-erbB-2) (neu proto-oncogene) (tyrosine kinase-type cell surface receptor HER2) (MLN 19) (CD340 antigen)_ERBB2_870_882_P04626	2.73*E* − 06	0.00024328
T-cell surface glycoprotein CD3 epsilon chain, T-cell surface antigen T3/Leu-4 epsilon chain, CD3e_CD3E_182_194_P07766	2.84*E* − 06	0.00025288
3-Phosphoinositide-dependent protein kinase 1 (EC 2.7.11.1) (hPDK1)_PDPK1_369_381_O15530	3.02*E* − 06	0.00026873
Hepatocyte growth factor receptor precursor (EC 2.7.10.1) (HGF receptor) (scatter factor receptor) (SF receptor) (HGF/SF receptor) (Met proto-oncogene tyrosine kinase) (c-Met)_MET_1227_1239_P08581	3.42*E* − 06	0.0003046
Ras GTPase-activating protein 1 (GTPase-activating protein) (GAP) (Rasp21 protein activator) (p120GAP) (RasGAP)_RASA1_453_465_P20936	4.01*E* − 06	0.00035652
B- and T-lymphocyte attenuator, B- and T-lymphocyte-associated protein, CD272_BTLA_252_262_Q7Z6A9	4.20*E* − 06	0.00037375
40S ribosomal protein S6 (phosphoprotein NP33)_RS6_228_240P62753	1.57*E* − 05	0.00139465
Early E1A 32 kDa protein_E1A_ADE05_212_224P03255	2.12*E* − 05	0.00188822
Tyrosine-protein kinase JAK3 (EC:2.7.10.2) (janus kinase 3) (JAK-3)_JAK3_974_986_P52333	2.20*E* − 05	0.00196214
cAMP-dependent protein kinase type II-beta regulatory subunit_KAP3_107_119P31323	2.76*E* − 05	0.00245874
Insulin receptor substrate 1_IRS1_890_902_P35568	3.07*E* − 05	0.00272913
Ephrin type-A receptor 2 precursor (EC 2.7.10.1) (tyrosine-protein kinase receptor ECK) (epithelial cell kinase)_EPHA2_765_777_P29317	7.62*E* − 05	0.00677916
Proto-oncogene tyrosine-protein kinase LCK (EC 2.7.10.2) (p56-LCK) (lymphocyte cell-specific protein-tyrosine kinase) (LSK) (T-cell-specific protein-tyrosine kinase)_LCK_387_399_P06239	0.00012233	0.01088757
T-cell surface glycoprotein CD3 zeta chain precursor (T-cell receptor T3 zeta chain) (CD247 antigen)_CD3Z_77_89_P20963	0.00013128	0.01168433
Protein 4.1 (Band 4.1) (P4.1) (EPB4.1) (4.1 R)_41_654_666_P11171	0.0001331	0.01184597
Pleckstrin (platelet p47 protein)_PLEK_106_118P08567	0.00013359	0.01188922
Tyrosine-protein kinase ZAP-70 (EC 2.7.10.2) (70 kDa zeta-associated protein) (syk-related tyrosine kinase)_ZAP70_485_497_P43403	0.00014176	0.01261638
Ephrin type-A receptor 1 precursor (EC 2.7.10.1) (tyrosine-protein kinase receptor EPH)_EPHA1_774_786_P21709	0.00019104	0.01700258
Glycogen synthase kinase-3 beta (EC:2.7.11.26), serine/threonine-protein kinase GSK3B (EC:2.7.11.1)_GSK3B_210_222_C218S_P49841	0.00030404	0.0270594
Receptor tyrosine-protein kinase erbB-2 precursor (EC 2.7.10.1) (p185erbB2) (C-erbB-2) (neu proto-oncogene) (tyrosine kinase-type cell surface receptor HER2) (MLN 19) (CD340 antigen)_ERBB2_679_691P04626	0.00041011	0.0364998
RAF proto-oncogene serine/threonine-protein kinase (EC 2.7.11.1) (Raf-1) (C-RAF) (cRaf)_RAF1_332_344_P04049	0.00051147	0.04552087

## Data Availability

The data used to support the findings of this study are available from the corresponding author upon request.

## Supplementary Materials

Supplementary Materials Suppl. Figure 1: MAPK signaling pathway of A: NCI-H2502, B: NCI-H2452, C: MSTO-211H, and D: MRC-5. Suppl. Figure 2: cell cycle pathway of A: NCI-H2502, B: NCI-H2452, C: MSTO-211H, and D: MRC5. Suppl. Figure 3: cancer pathways of A: NCI-H2502, B: NCI-H2452, C: MSTO-211H, and D: MRC-5. Suppl. Figure 4: phosphorylation level of A: ARAF, B: EPHA1, C: EPHA2, and D: EPHA7 in all cell lines. For each cell line, phosphorylation levels are depicted before (medium) and after cisplatin treatment (Cis). Suppl. Figure 5: phosphorylation level of A: KIT, B: PTPN11, C: PIK3R1, and D: PTPN6 in all cell lines. For each cell line, phosphorylation levels are depicted before (medium) and after cisplatin treatment (Cis). Suppl. Figure 6: phosphorylation level of A: KDR, B: EFS, C: AKT1, and D: PTK2B/FAK2 in all cell lines. For each cell line, phosphorylation levels are depicted before (medium) and after cisplatin treatment (Cis). Suppl. Figure 7: score plots and volcano plots of PTK upstream kinase analysis: A: score plot of PTK upstream kinase analysis for NCI-H2052 cells. B: volcano plot of PTK-upstream kinase analysis for NCI-H2052 cells. C: score plot of PTK-upstream kinase analysis for NCI-H2452 cells. D: volcano plot of PTK-upstream kinase analysis for NCI-H2452 cells. E: score plot of PTK-upstream kinase analysis for MSTO-211H cells. F: volcano plot of PTK-upstream kinase analysis for MSTO211H cells. G: score plot of PTK-upstream kinase analysis for MRC-5 cells. H: volcano plot of PTK upstream kinase analysis for MRC-5 cells. Suppl. Figure 8: score plots and volcano plots of STK upstream kinase analysis: A: score plot of STK upstream kinase analysis for NCI-H2052 cells. B: volcano plot of STK upstream kinase analysis for NCI-H2052 cells. C: score plot of STK upstream kinase analysis for NCI-H2452 cells. D: volcano plot of STK upstream kinase analysis for NCI-H2452 cells. E: score plot of STK upstream kinase analysis for MSTO-211H cells. F: volcano plot of STK upstream kinase analysis for MSTO211H cells. G: score plot of STK upstream kinase analysis for MRC-5 cells. H: volcano plot of STK upstream kinase analysis for MRC-5 cells. Suppl. Figure 9: kinase-tree of A: NCI-H2052, B: MSTO-211H, C: NCI-H2452, and D: MRC-5. Suppl. Table 1: *p* values of the influenced phosphosites during cisplatin therapy. Suppl. Table 2: significantly altered phosphorylation levels between the different cell lines. Suppl. Table 3: association of phosphosite phosphorylation (“+” = high and “−” = low phosphorylation) to apoptosis ratio of cells after cisplatin treatment.
