# Supplementary material for: Screening of Pleural Mesothelioma Cell Lines for Kinase Activity May Identify New Mechanisms of Therapy Resistance in Patients Receiving Platin-Based Chemotherapy
Source: J Oncol. 2019 Dec 23;2019:2902985. doi: 10.1155/2019/2902985 (PMC6942867; doi:10.1155/2019/2902985)
Supplement: Supplementary Materials — Suppl. Figure 1: MAPK signaling pathway of A: NCI-H2502, B: NCI-H2452, C: MSTO-211H, and D: MRC-5. Suppl. Figure 2: cell cycle pathway of A: NCI-H2502, B: NCI-H2452, C: MSTO-211H, and D: MRC5. Suppl. Figure 3: cancer pathways of A: NCI-H2502, B: NCI-H2452, C: MSTO-211H, and D: MRC-5. Suppl. Figure 4: phosphorylation level of A: ARAF, B: EPHA1, C: EPHA2, and D: EPHA7 in all cell lines. For each cell line, phosphorylation levels are depicted before (medium) and after cisplatin treatment (Cis). Suppl. Figure 5: phosphorylation level of A: KIT, B: PTPN11, C: PIK3R1, and D: PTPN6 in all cell lines. For each cell line, phosphorylation levels are depicted before (medium) and after cisplatin treatment (Cis). Suppl. Figure 6: phosphorylation level of A: KDR, B: EFS, C: AKT1, and D: PTK2B/FAK2 in all cell lines. For each cell line, phosphorylation levels are depicted before (medium) and after cisplatin treatment (Cis). Suppl. Figure 7: score plots and volcano plots of PTK upstream kinase analysis: A: score plot of PTK upstream kinase analysis for NCI-H2052 cells. B: volcano plot of PTK-upstream kinase analysis for NCI-H2052 cells. C: score plot of PTK-upstream kinase analysis for NCI-H2452 cells. D: volcano plot of PTK-upstream kinase analysis for NCI-H2452 cells. E: score plot of PTK-upstream kinase analysis for MSTO-211H cells. F: volcano plot of PTK-upstream kinase analysis for MSTO211H cells. G: score plot of PTK-upstream kinase analysis for MRC-5 cells. H: volcano plot of PTK upstream kinase analysis for MRC-5 cells. Suppl. Figure 8: score plots and volcano plots of STK upstream kinase analysis: A: score plot of STK upstream kinase analysis for NCI-H2052 cells. B: volcano plot of STK upstream kinase analysis for NCI-H2052 cells. C: score plot of STK upstream kinase analysis for NCI-H2452 cells. D: volcano plot of STK upstream kinase analysis for NCI-H2452 cells. E: score plot of STK upstream kinase analysis for MSTO-211H cells. F: volcano plot of STK upstream kinase anal [file 2902985.f1.pdf]

Supplementary

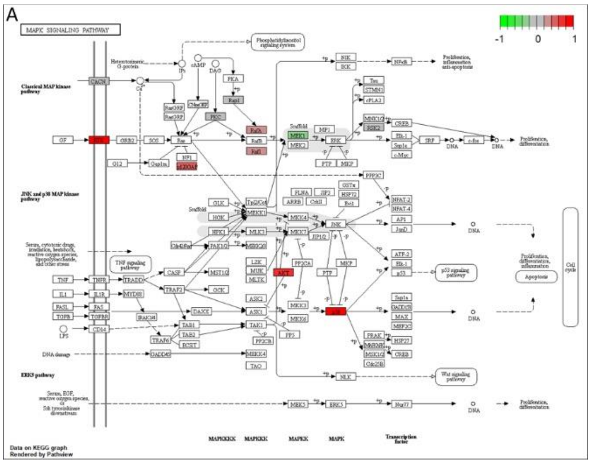

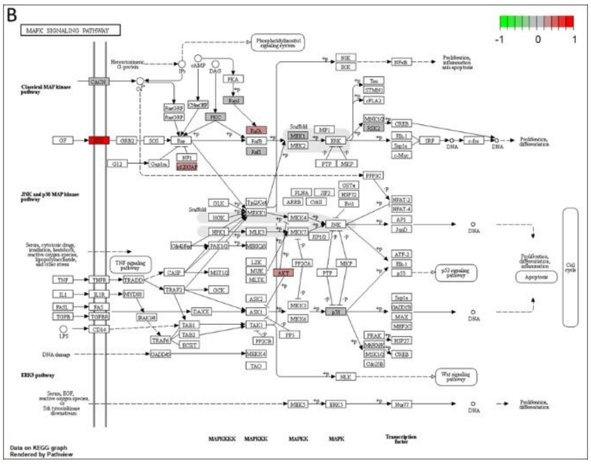

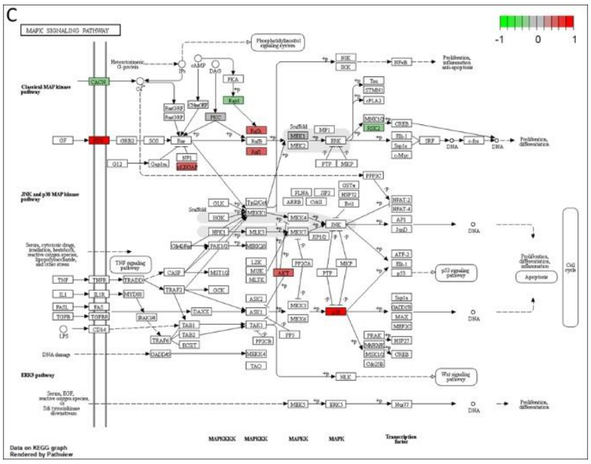

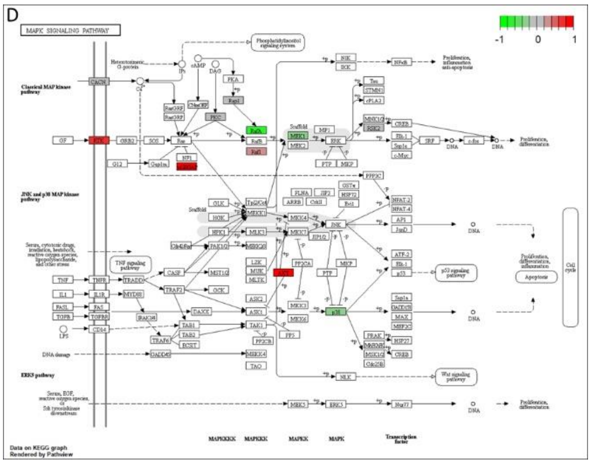

Suppl. Figure 1: MAPK signalling pathway of A: NCI-H2502, B: NCI-H2452, C: MSTO-211H and D: MRC-5.

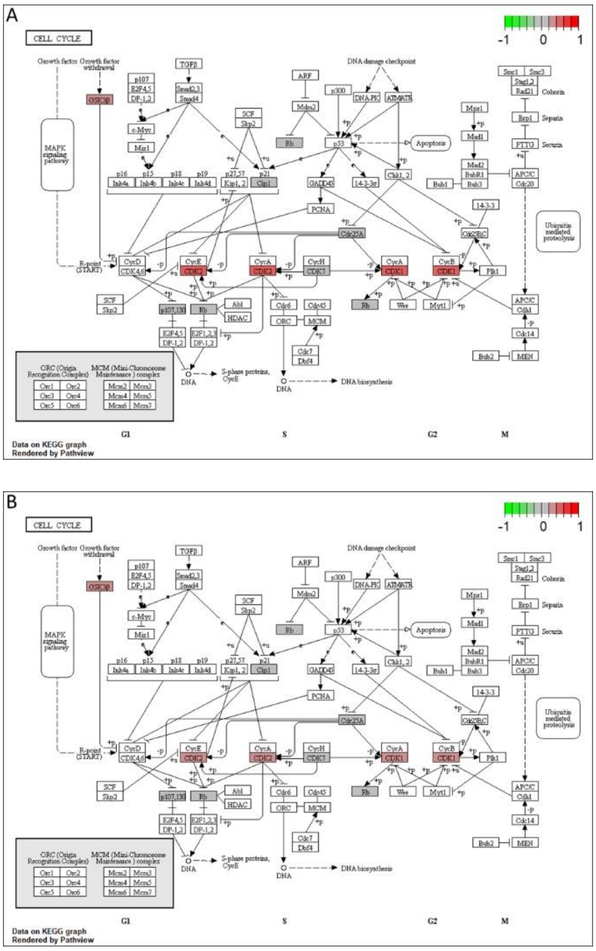

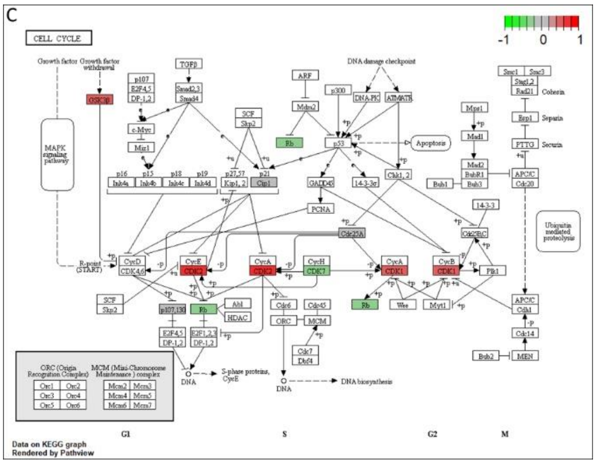

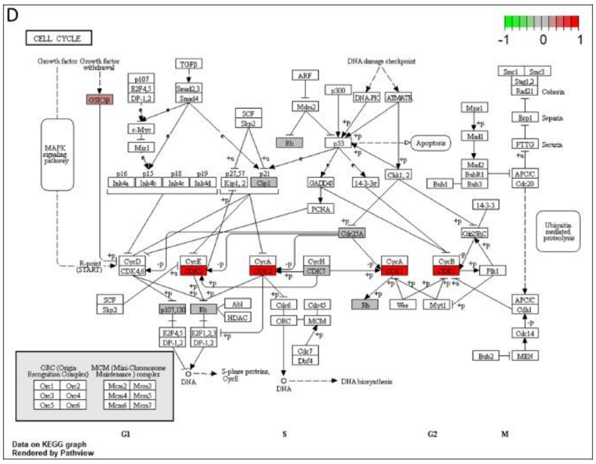

Suppl. Figure 2: Cell cycle pathway of A: NCI-H2502, B: NCI-H2452, C: MSTO-211H and D: MRC-5.

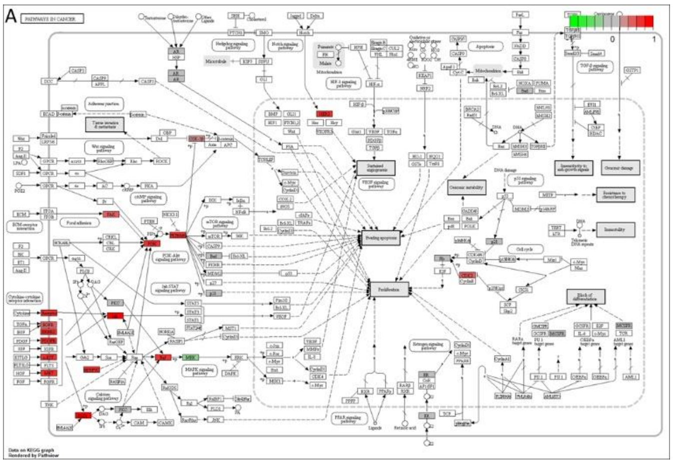

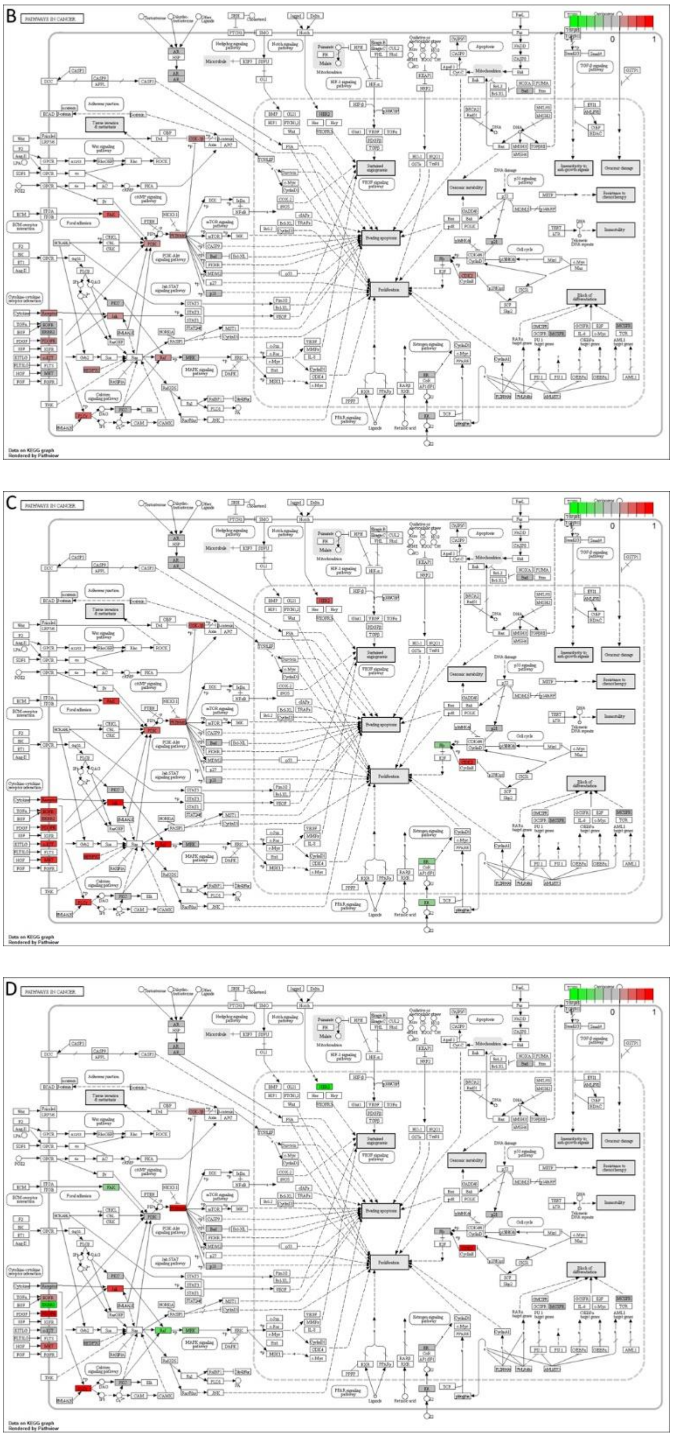

Suppl. Figure 3: Cancer pathways of A: NCI-H2502, B: NCI-H2452, C: MSTO-211H and D: MRC-5.

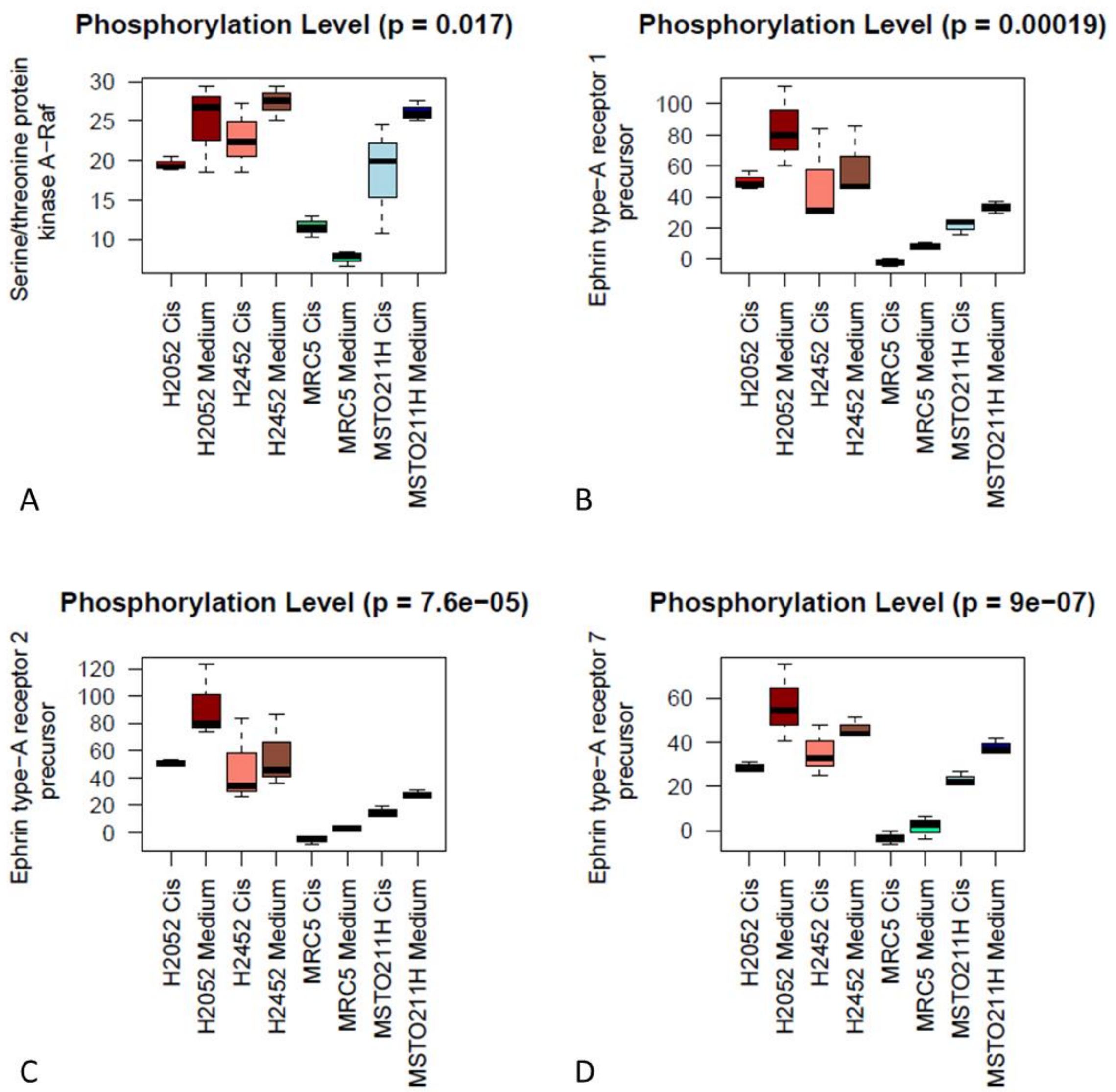

Suppl. Figure 4: Phosphorylation level of **A**: ARAF, **B**: EPHA1, **C**: EPHA2, **D**: EPHA7 in all cell lines. For each cell line, phosphorylation levels are depicted before (Medium) and after cisplatin treatment (Cis).

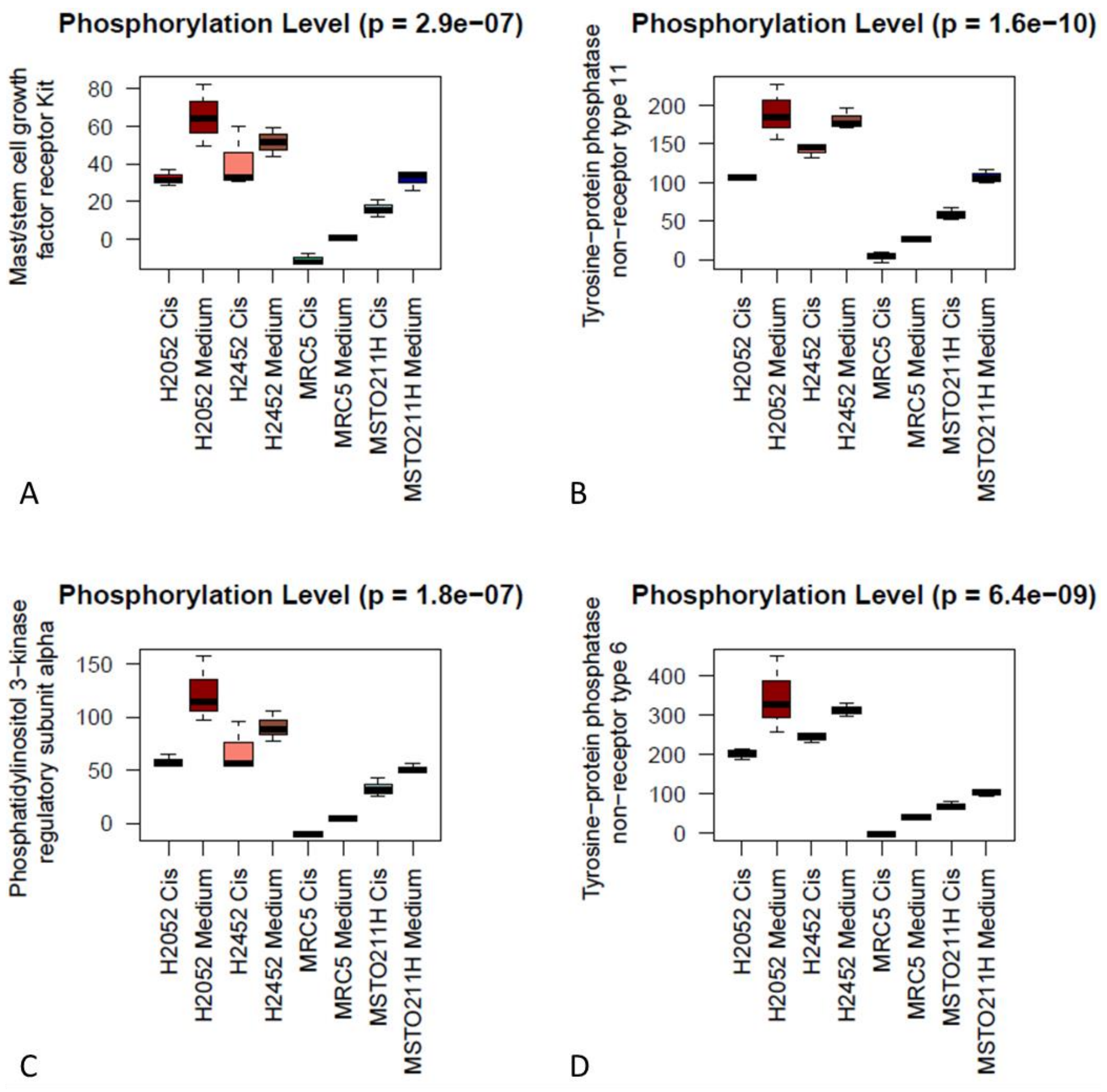

Suppl. Figure 5: Phosphorylation Level of **A**: KIT, **B**: PTPN11, **C**: PIK3R1, **D**: PTPN6 in all cell lines. For each cell line, phosphorylation levels are depicted before (Medium) and after cisplatin treatment (Cis).

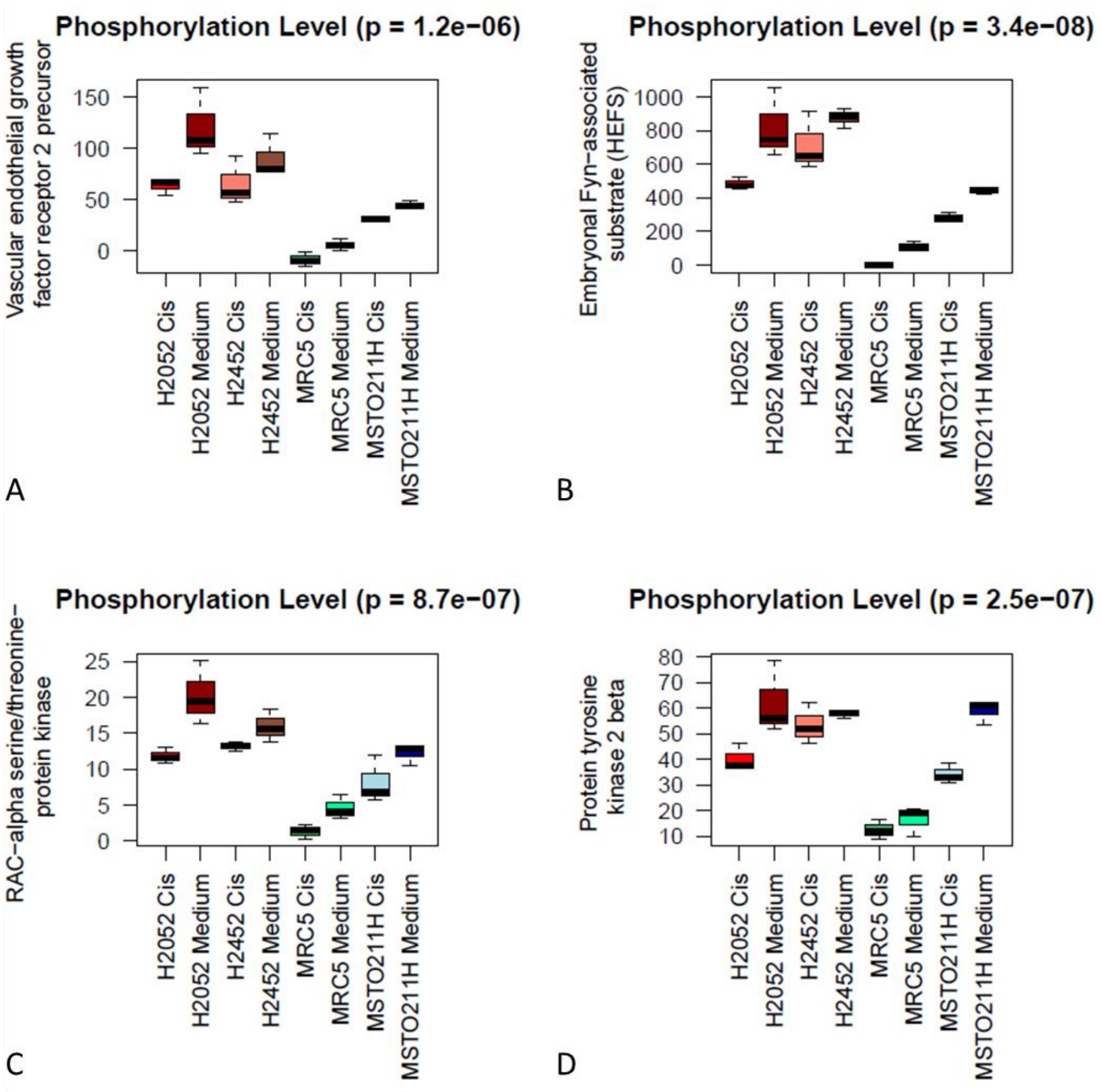

Suppl. Figure 6: Phosphorylation Level of **A**: KDR, **B**: EFS, **C**: AKT1, **D**: PTK2B/FAK2 in all cell lines. For each cell line, phosphorylation levels are depicted before (Medium) and after cisplatin treatment (Cis).

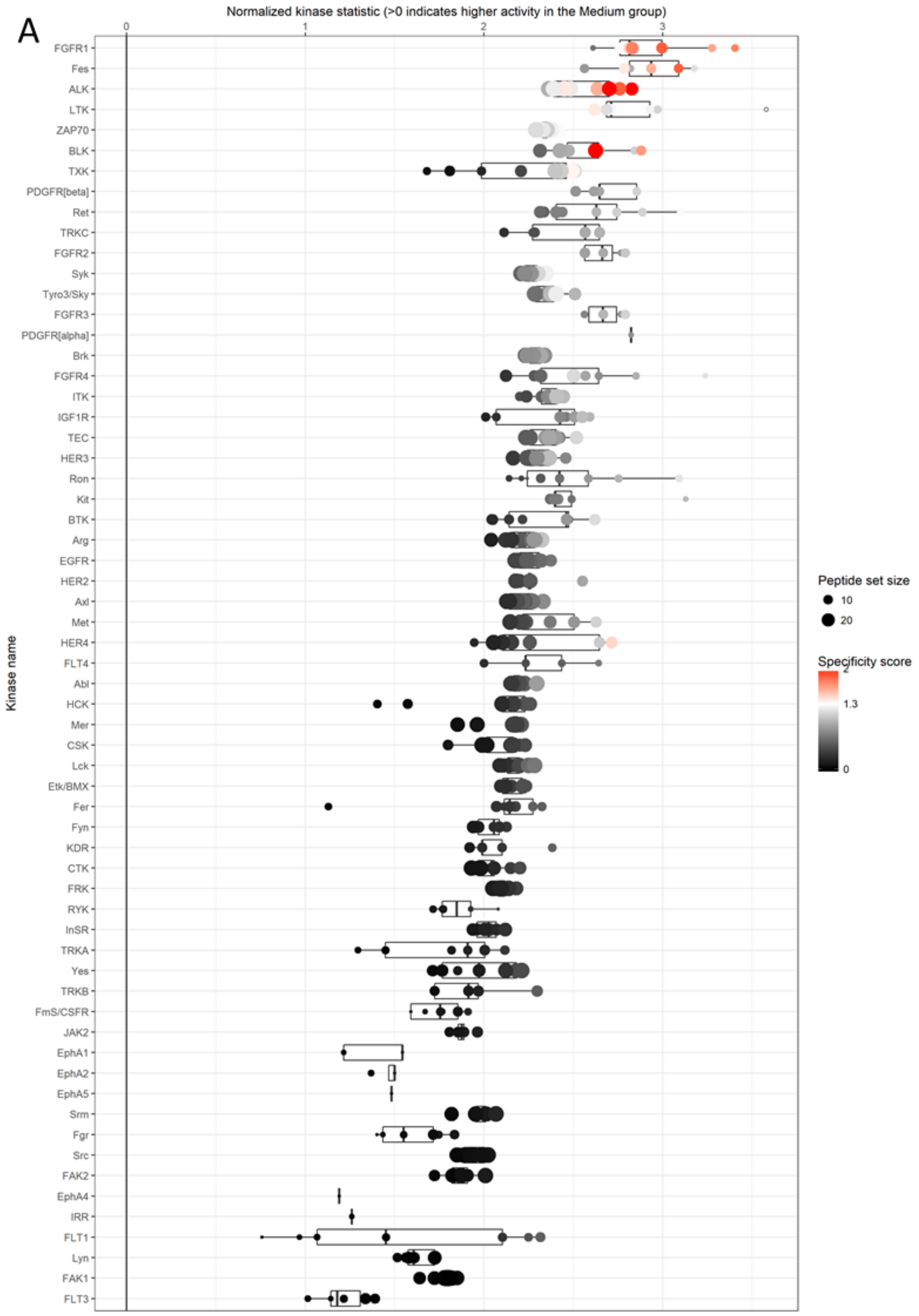
A
Normalized kinase statistic (>0 indicates higher activity in the Medium group)
0
1
2
3
Kinase name
FGFR1
Fes
ALK
LTK
ZAP70
BLK
TXK
PDGFR[beta]
Ret
TRKC
FGFR2
Syk
Tyro3/Sky
FGFR3
PDGFR[alpha]
Brk
FGFR4
ITK
IGF1R
TEC
HER3
Ron
Kit
BTK
Arg
EGFR
HER2
Axl
Met
HER4
FLT4
Abl
HCK
Mer
CSK
Lck
Etk/BMX
Fer
Fyn
KDR
CTK
FRK
RYK
InSR
TRKA
Yes
TRKB
FmS/CSFR
JAK2
EphA1
EphA2
EphA5
Srm
Fgr
Src
FAK2
EphA4
IRR
FLT1
Lyn
FAK1
FLT3
Peptide set size
10
20
Specificity score
2
1.3
0

B

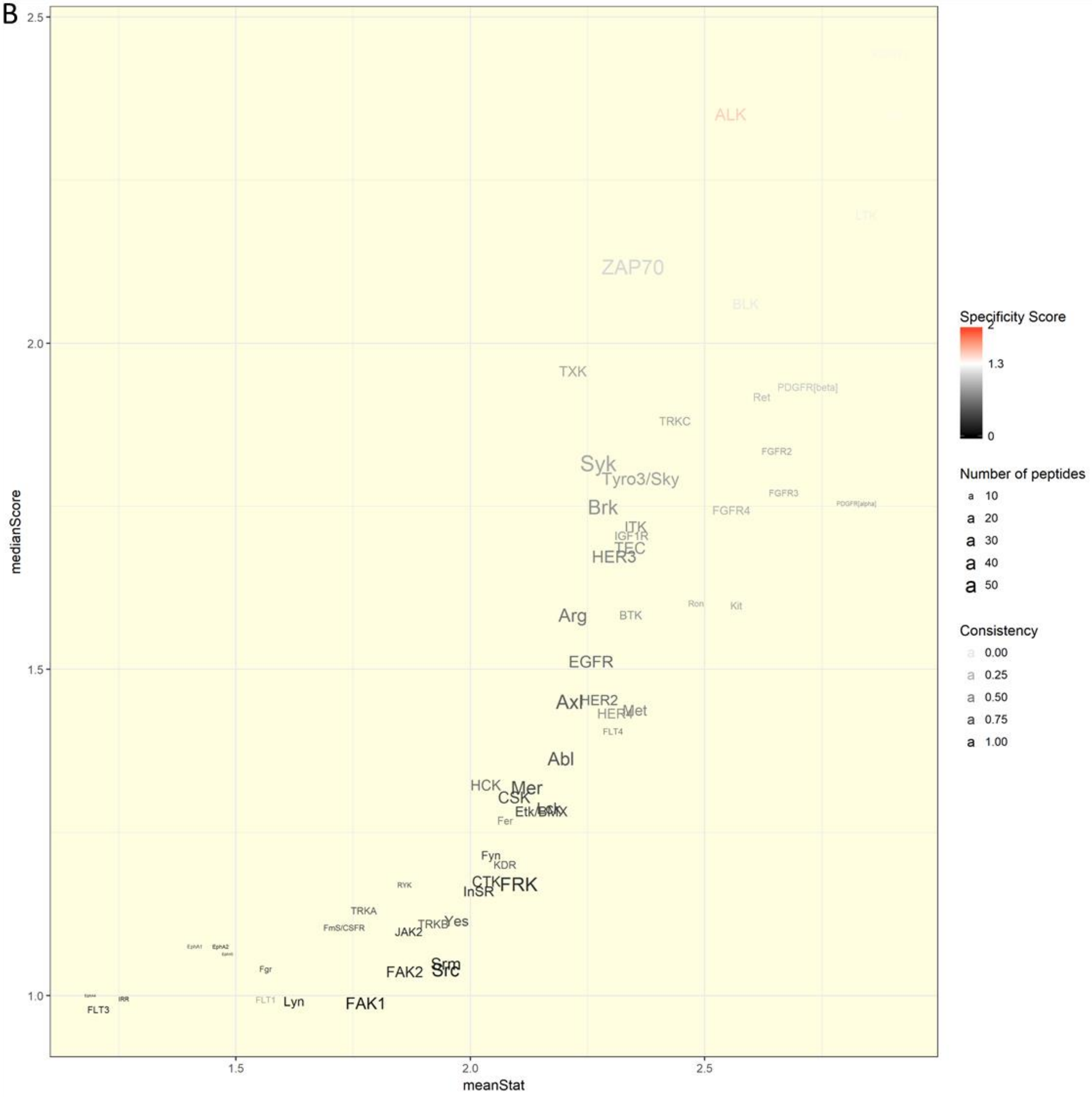

medianScore
meanStat
2.5
2.0
1.5
1.0
ALK
LTK
ZAP70
BLK
TXK
Ret
PDGFR[beta]
TRKC
FGFR2
Syk
Tyro3/Sky
FGFR3
Brk
FGFR4
ITK
IGF1R
TEC
HER3
Ron
Kit
Arg
BTK
EGFR
Axl
HER2
HER4
Met
FLT4
Abl
HCK
Mer
CSK
Lck
Etk/BMX
Fer
Fyn
KDR
CTK
FRK
InSR
RYK
TRKA
FmS/CSFR
TRKB
Yes
JAK2
EphA1
EphA2
Fgr
FAK2
Srm
Src
IRR
FLT3
FLT1
Lyn
FAK1
Specificity Score
2
1.3
0
Number of peptides
10
20
30
40
50
Consistency
0.00
0.25
0.50
0.75
1.00

C

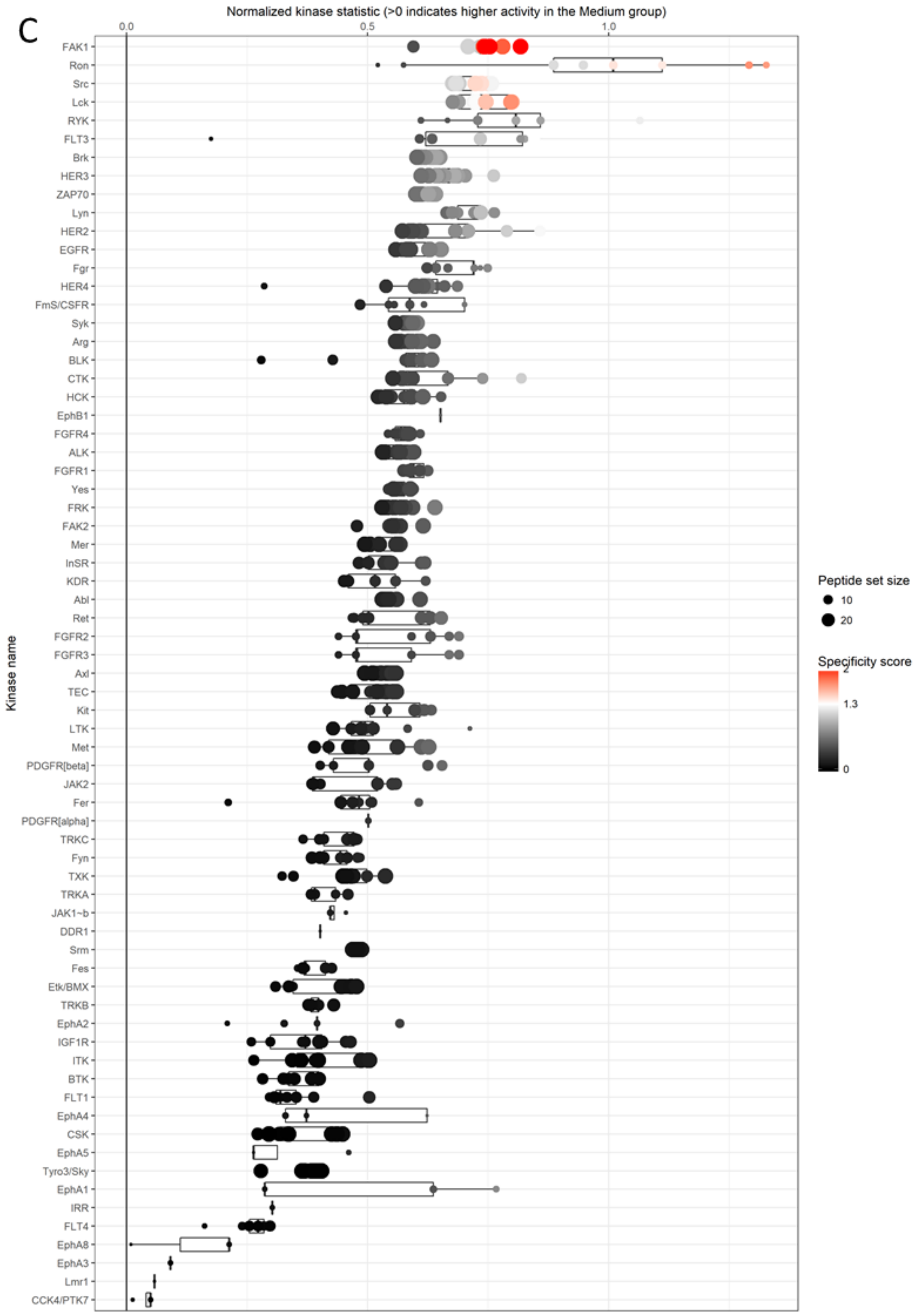

Normalized kinase statistic (>0 indicates higher activity in the Medium group)
0.0
0.5
1.0
Kinase name
FAK1
Ron
Src
Lck
RYK
FLT3
Brk
HER3
ZAP70
Lyn
HER2
EGFR
Fgr
HER4
FmS/CSFR
Syk
Arg
BLK
CTK
HCK
EphB1
FGFR4
ALK
FGFR1
Yes
FRK
FAK2
Mer
InSR
KDR
Abl
Ret
FGFR2
FGFR3
Axl
TEC
Kit
LTK
Met
PDGFR[beta]
JAK2
Fer
PDGFR[alpha]
TRKC
Fyn
TXK
TRKA
JAK1~b
DDR1
Srm
Fes
Etk/BMX
TRKB
EphA2
IGF1R
ITK
BTK
FLT1
EphA4
CSK
EphA5
Tyro3/Sky
EphA1
IRR
FLT4
EphA8
EphA3
Lmr1
CCK4/PTK7
Peptide set size
10
20
Specificity score
2
1.3
0

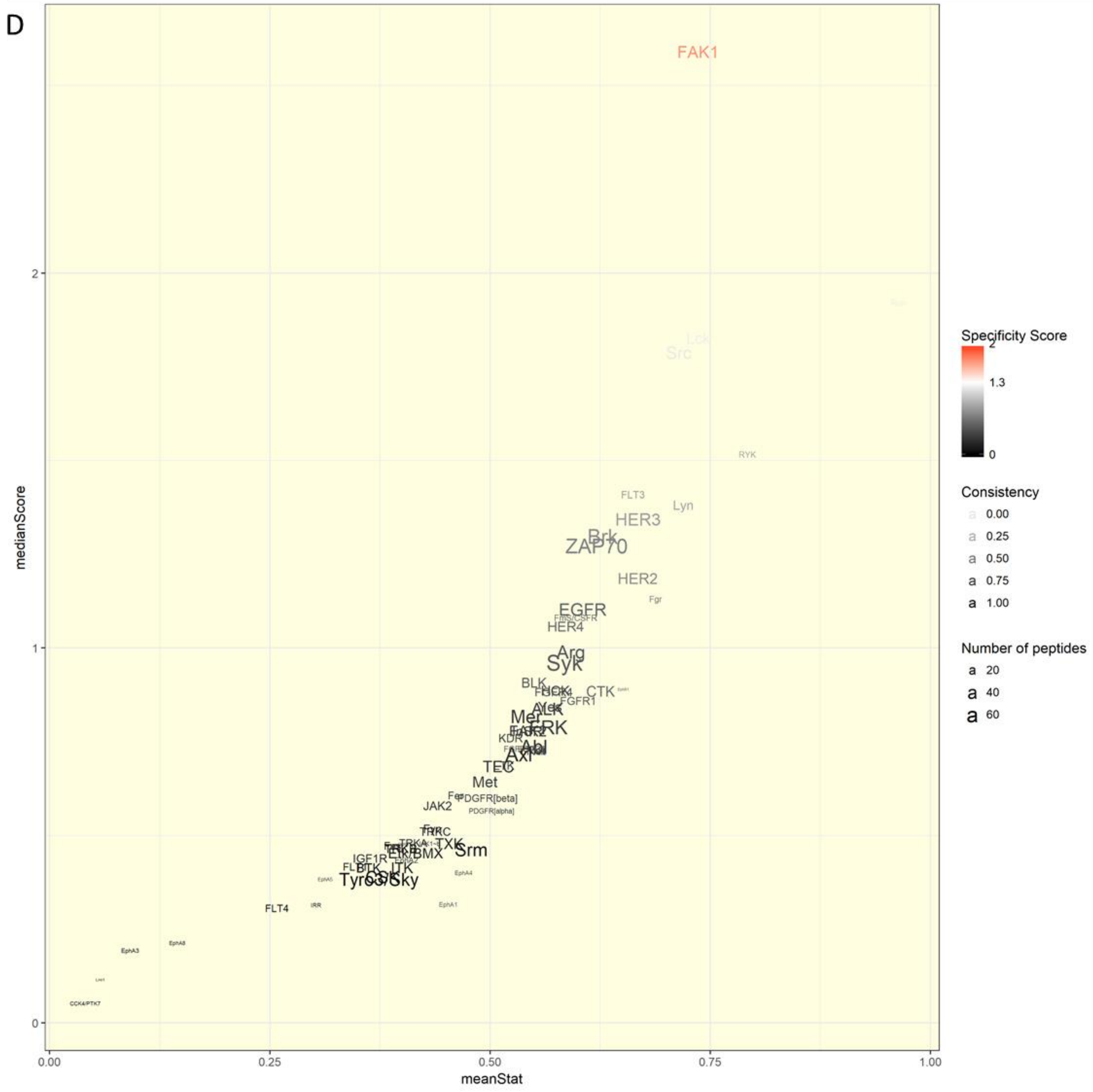
D
FAK1
Lck
Src
RYK
FLT3
Lyn
HER3
Brk
ZAP70
HER2
Fgr
EGFR
HER4
Arg
Syk
BLK
CTK
FGFR1
Mer
ALK
FRK
KDR
Abl
Axl
TEC
Met
JAK2
PDGFR[beta]
PDGFR[alpha]
TRKC
TXK
Srm
BMX
IGF1R
BTK
ITK
Tyro3/Sky
EphA4
FLT4
IRR
EphA1
EphA3
EphA8
CCK4/PTK7
medianScore
meanStat
0
1
2
0.00
0.25
0.50
0.75
1.00
Specificity Score
2
1.3
0
Consistency
0.00
0.25
0.50
0.75
1.00
Number of peptides
20
40
60

E

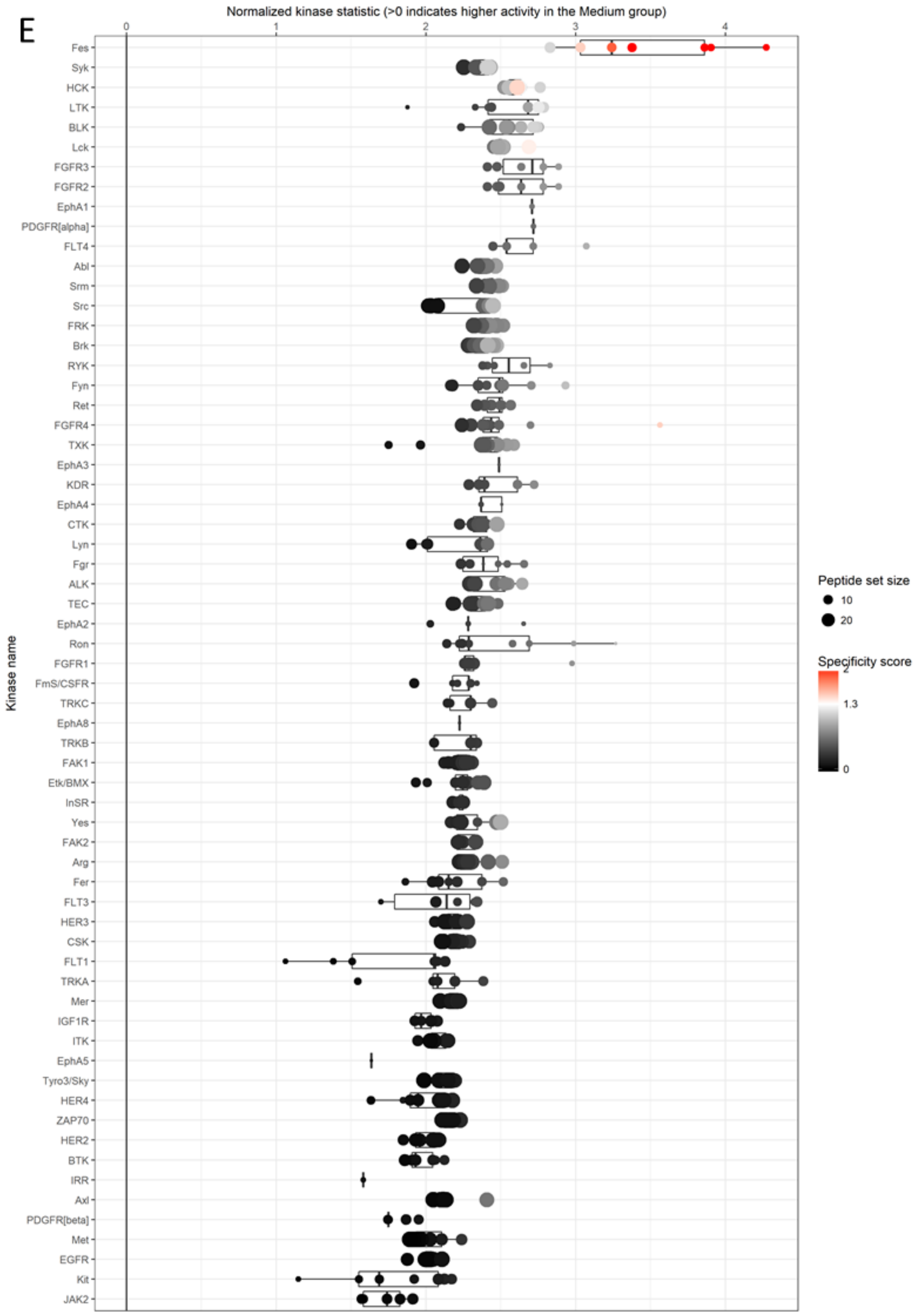
Normalized kinase statistic (>0 indicates higher activity in the Medium group)
0
1
2
3
4
Kinase name
Fes
Syk
HCK
LTK
BLK
Lck
FGFR3
FGFR2
EphA1
PDGFR[alpha]
FLT4
Abl
Srm
Src
FRK
Brk
RYK
Fyn
Ret
FGFR4
TXK
EphA3
KDR
EphA4
CTK
Lyn
Fgr
ALK
TEC
EphA2
Ron
FGFR1
FmS/CSFR
TRKC
EphA8
TRKB
FAK1
Etk/BMX
InSR
Yes
FAK2
Arg
Fer
FLT3
HER3
CSK
FLT1
TRKA
Mer
IGF1R
ITK
EphA5
Tyro3/Sky
HER4
ZAP70
HER2
BTK
IRR
Axl
PDGFR[beta]
Met
EGFR
Kit
JAK2
Peptide set size
10
20
Specificity score
2
1.3
0

F

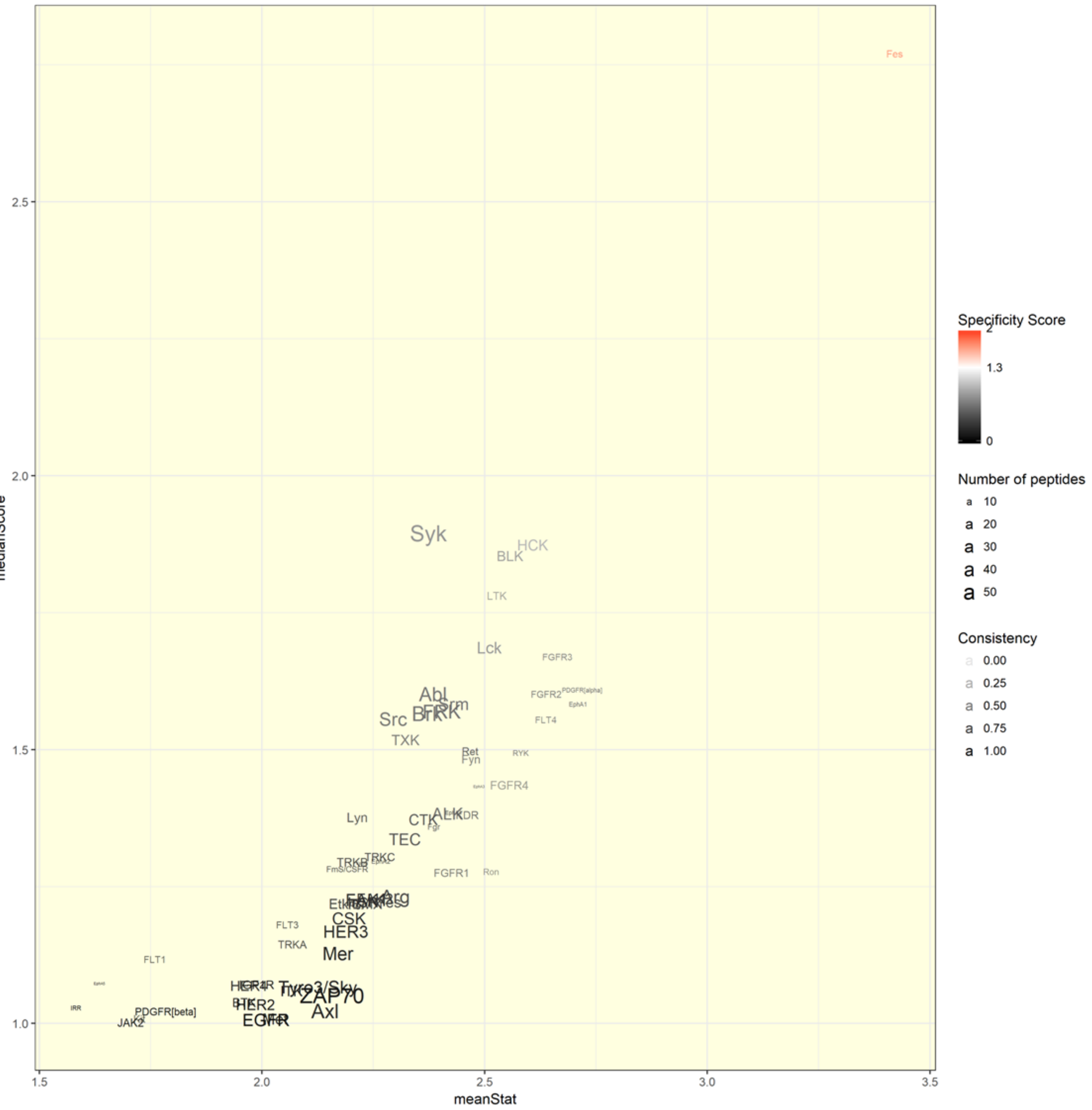

Fes
Syk
HCK
BLK
LTK
Lck
FGFR3
Abl
FGFR2
PDGFR[alpha]
EphA1
Src
Srm
FLT4
TXK
Ret
Fyn
RYK
FGFR4
Lyn
CTK
ALK
KDR
TEC
TRKC
TRKB
FmS/CSFR
FGFR1
Ron
Arg
CSK
FLT3
HER3
TRKA
Mer
FLT1
HER4
Tyro3/Sky
ZAP70
IRR
HER2
PDGFR[beta]
Axl
JAK2
EGFR
medianScore
meanStat
Specificity Score
Number of peptides
Consistency

G

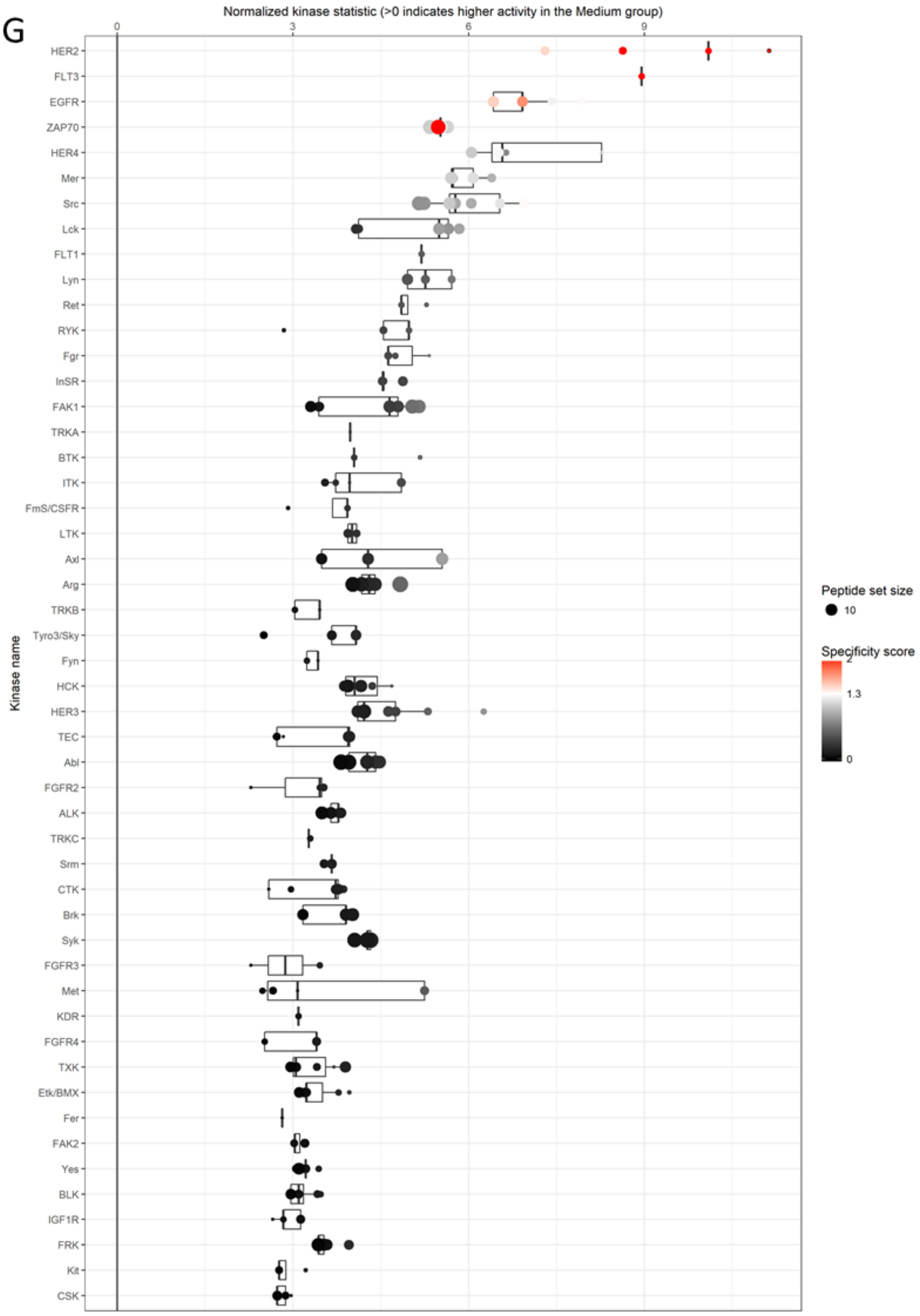
Normalized kinase statistic (>0 indicates higher activity in the Medium group)
0
3
6
9
Kinase name
HER2
FLT3
EGFR
ZAP70
HER4
Mer
Src
Lck
FLT1
Lyn
Ret
RYK
Fgr
InSR
FAK1
TRKA
BTK
ITK
FmS/CSFR
LTK
Axl
Arg
TRKB
Tyro3/Sky
Fyn
HCK
HER3
TEC
Abl
FGFR2
ALK
TRKC
Srm
CTK
Brk
Syk
FGFR3
Met
KDR
FGFR4
TXK
Etk/BMX
Fer
FAK2
Yes
BLK
IGF1R
FRK
Kit
CSK
Peptide set size
10
Specificity score
2
1.3
0

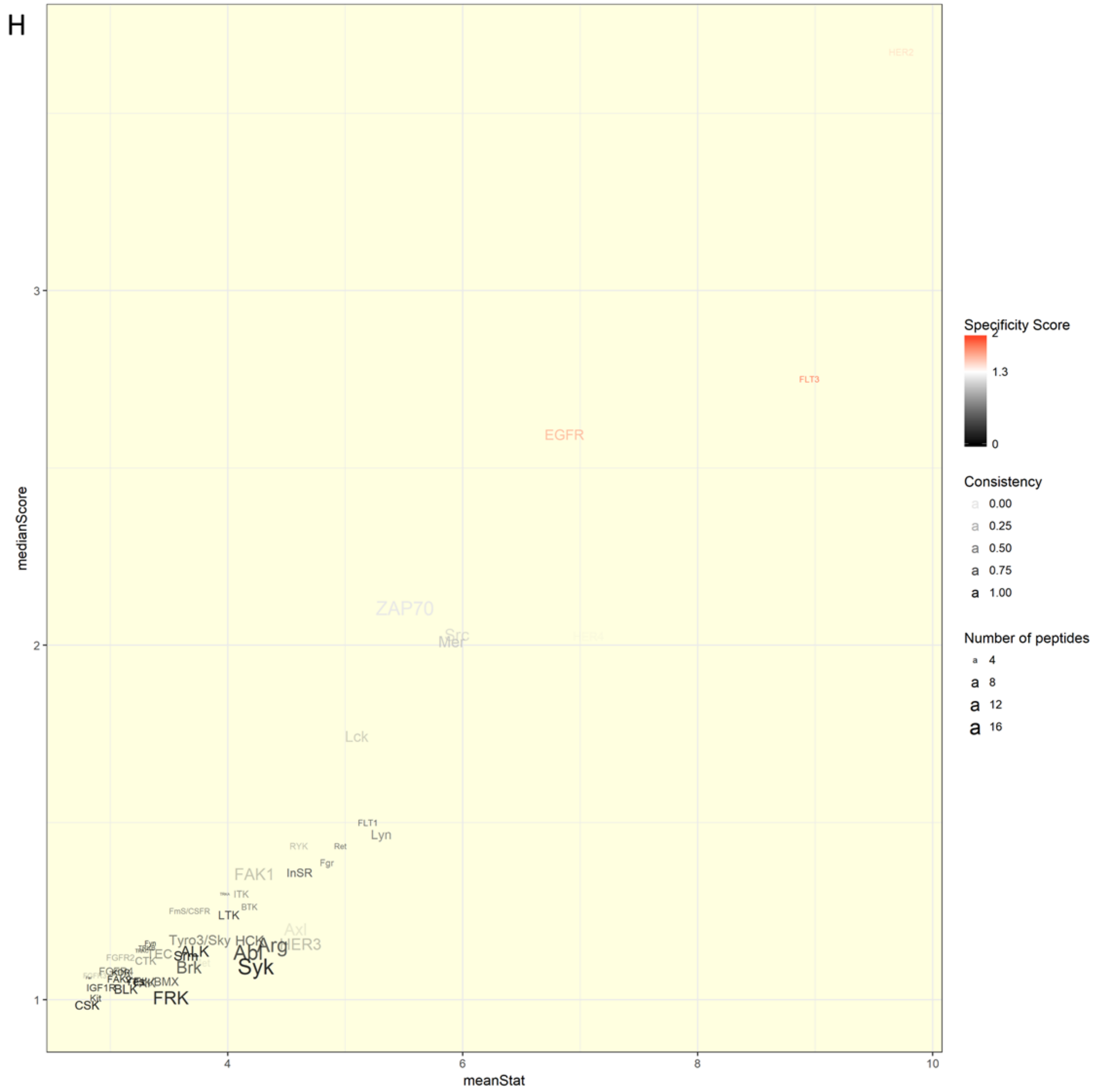

Suppl. Figure 7: Scoreplots and volcanoplots of PTK upstream kinase analysis: A: Scoreplot of PTK-Upstream kinase analysis for NCI-H2052 cells. B: Volcanoplot of PTK-Upstream kinase analysis for NCI-H2052 cells. C: Scoreplot of PTK-Upstream kinase analysis for NCI-H2452 cells. D: Volcanoplot of PTK-Upstream kinase analysis for NCI-H2452 cells. E: Scoreplot of PTK-Upstream kinase analysis for MSTO-211H cells. F: Volcanoplot of PTK-Upstream kinase analysis for MSTO-211H cells. G: Scoreplot of PTK-Upstream kinase analysis for MRC-5cells. H: Volcanoplot of PTK-Upstream kinase analysis for MRC-5 cells.

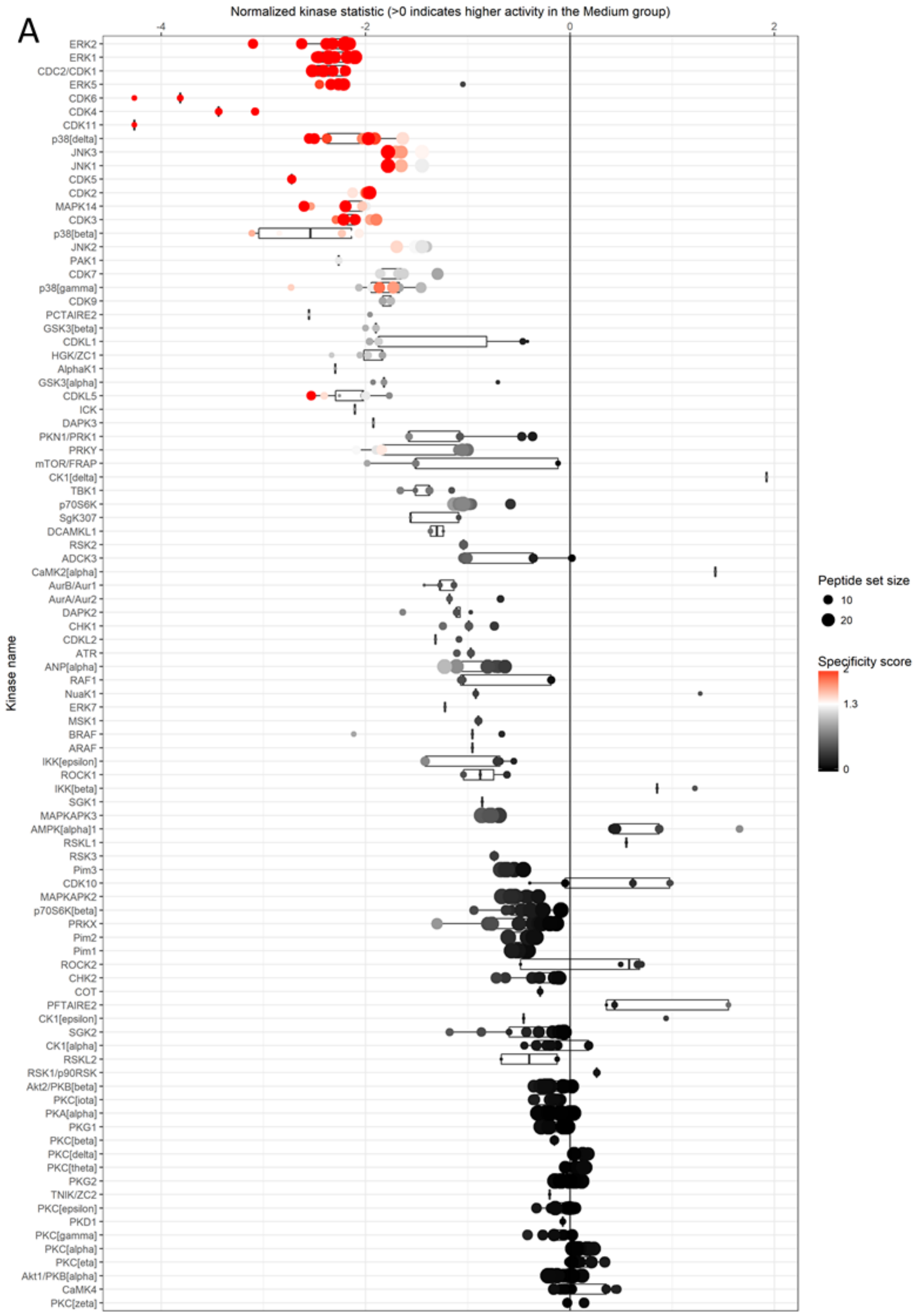
A
Normalized kinase statistic (>0 indicates higher activity in the Medium group)
-4
-2
0
2
Kinase name
ERK2
ERK1
CDC2/CDK1
ERK5
CDK6
CDK4
CDK11
p38[delta]
JNK3
JNK1
CDK5
CDK2
MAPK14
CDK3
p38[beta]
JNK2
PAK1
CDK7
p38[gamma]
CDK9
PCTAIRE2
GSK3[beta]
CDKL1
HGK/ZC1
AlphaK1
GSK3[alpha]
CDKL5
ICK
DAPK3
PKN1/PRK1
PRKY
mTOR/FRAP
CK1[delta]
TBK1
p70S6K
SgK307
DCAMKL1
RSK2
ADCK3
CaMK2[alpha]
AurB/Aur1
AurA/Aur2
DAPK2
CHK1
CDKL2
ATR
ANP[alpha]
RAF1
NuaK1
ERK7
MSK1
BRAF
ARAF
IKK[epsilon]
ROCK1
IKK[beta]
SGK1
MAPKAPK3
AMPK[alpha]1
RSKL1
RSK3
Pim3
CDK10
MAPKAPK2
p70S6K[beta]
PRKX
Pim2
Pim1
ROCK2
CHK2
COT
PFTAIRE2
CK1[epsilon]
SGK2
CK1[alpha]
RSKL2
RSK1/p90RSK
Akt2/PKB[beta]
PKC[iota]
PKA[alpha]
PKG1
PKC[beta]
PKC[delta]
PKC[theta]
PKG2
TNIK/ZC2
PKC[epsilon]
PKD1
PKC[gamma]
PKC[alpha]
PKC[eta]
Akt1/PKB[alpha]
CaMK4
PKC[zeta]
Peptide set size
10
20
Specificity score
2
1.3
0

B

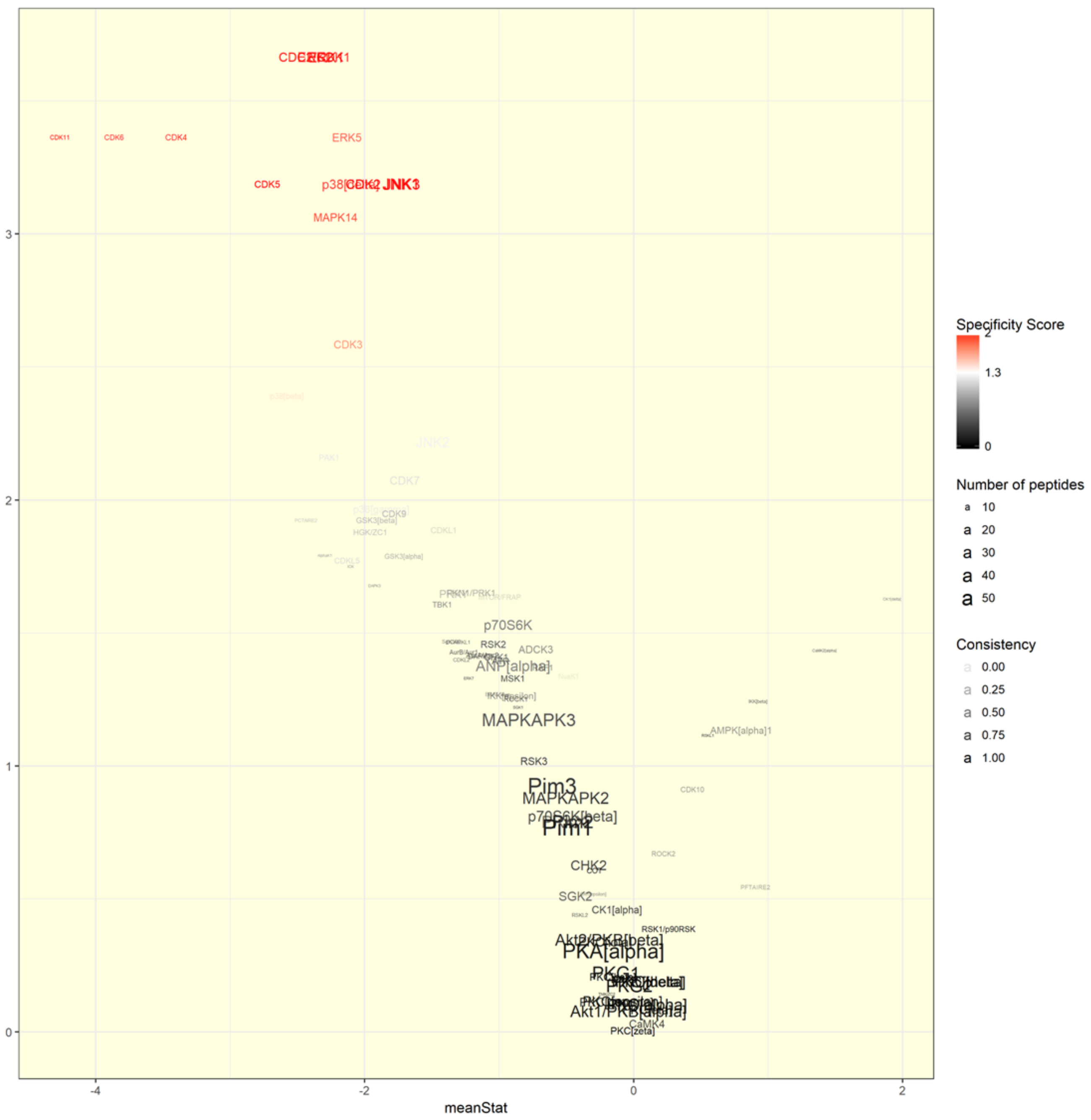

medianScore
meanStat
Specificity Score
2
1.3
0
Number of peptides
10
20
30
40
50
Consistency
0.00
0.25
0.50
0.75
1.00
CDK11
CDK6
CDK4
ERK5
CDK5
MAPK14
CDK3
JNK2
PAK1
CDK7
CDK9
GSK3[beta]
HGK/ZC1
CDKL1
GSK3[alpha]
CDKL5
TBK1
p70S6K
RSK2
ADCK3
ANP[alpha]
MSK1
MAPKAPK3
AMPK[alpha]1
RSK3
CDK10
MAPKAPK2
ROCK2
CHK2
PFTAIRE2
SGK2
CK1[alpha]
RSK1/p90RSK
PKA[alpha]
PKG1
PKG2
Akt1/PKB[alpha]
CaMK4
PKC[zeta]
-4
-2
0
2
3
1

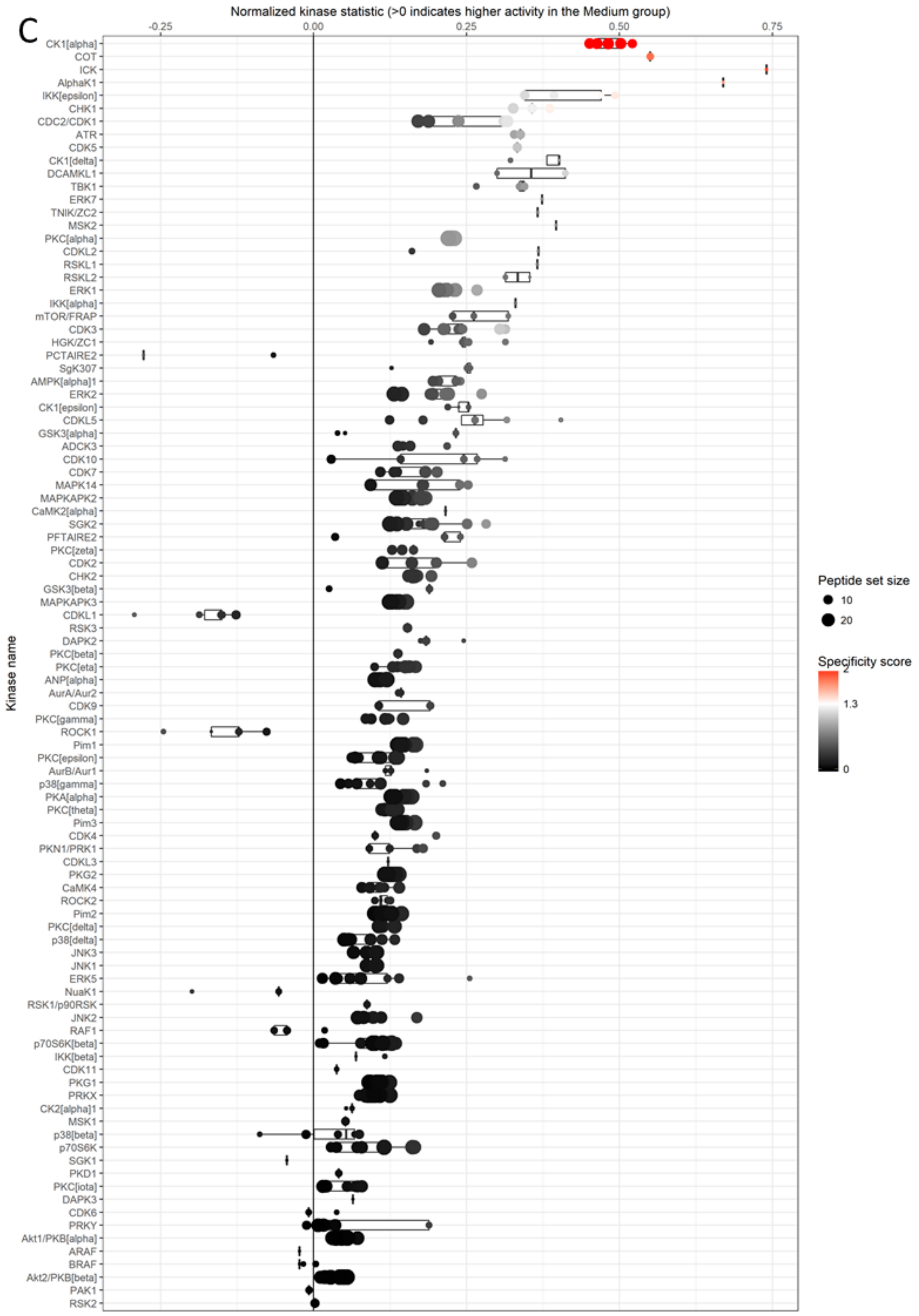
C
Normalized kinase statistic (>0 indicates higher activity in the Medium group)
-0.25
0.00
0.25
0.50
0.75
Kinase name
CK1[alpha]
COT
ICK
AlphaK1
IKK[epsilon]
CHK1
CDC2/CDK1
ATR
CDK5
CK1[delta]
DCAMKL1
TBK1
ERK7
TNIK/ZC2
MSK2
PKC[alpha]
CDKL2
RSKL1
RSKL2
ERK1
IKK[alpha]
mTOR/FRAP
CDK3
HGK/ZC1
PCTAIRE2
SgK307
AMPK[alpha]1
ERK2
CK1[epsilon]
CDKL5
GSK3[alpha]
ADCK3
CDK10
CDK7
MAPK14
MAPKAPK2
CaMK2[alpha]
SGK2
PFTAIRE2
PKC[zeta]
CDK2
CHK2
GSK3[beta]
MAPKAPK3
CDKL1
RSK3
DAPK2
PKC[beta]
PKC[eta]
ANP[alpha]
AurA/Aur2
CDK9
PKC[gamma]
ROCK1
Pim1
PKC[epsilon]
AurB/Aur1
p38[gamma]
PKA[alpha]
PKC[theta]
Pim3
CDK4
PKN1/PRK1
CDKL3
PKG2
CaMK4
ROCK2
Pim2
PKC[delta]
p38[delta]
JNK3
JNK1
ERK5
NuaK1
RSK1/p90RSK
JNK2
RAF1
p70S6K[beta]
IKK[beta]
CDK11
PKG1
PRKX
CK2[alpha]1
MSK1
p38[beta]
p70S6K
SGK1
PKD1
PKC[iota]
DAPK3
CDK6
PRKY
Akt1/PKB[alpha]
ARAF
BRAF
Akt2/PKB[beta]
PAK1
RSK2
Peptide set size
10
20
Specificity score
2
1.3
0

D

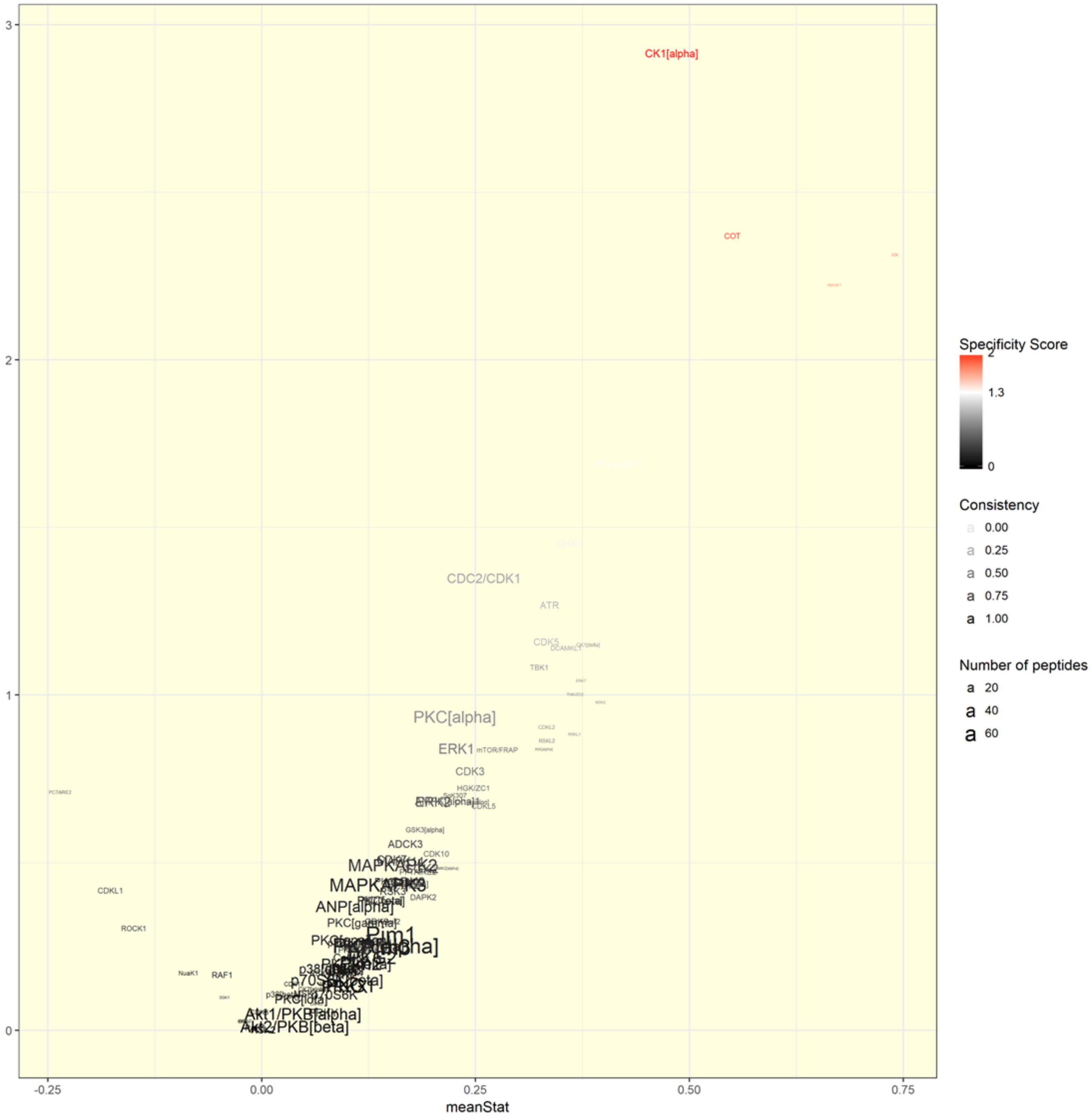

Specificity Score
2
1.3
0
Consistency
0.00
0.25
0.50
0.75
1.00
Number of peptides
20
40
60
CK1[alpha]
COT
CDC2/CDK1
ATR
CDK5
TBK1
PKC[alpha]
ERK1
mTOR/FRAP
CDK3
HGK/ZC1
GSK3[alpha]
ADCK3
CDK10
DAPK2
CDKL1
ROCK1
NuaK1
RAF1
Pim1
Akt1/PKB[alpha]
Akt2/PKB[beta]
PCTAIRE2
CDKL2
RSKL2
CDKL5
medianScore
meanStat
3
2
1
0
-0.25
0.00
0.25
0.50
0.75

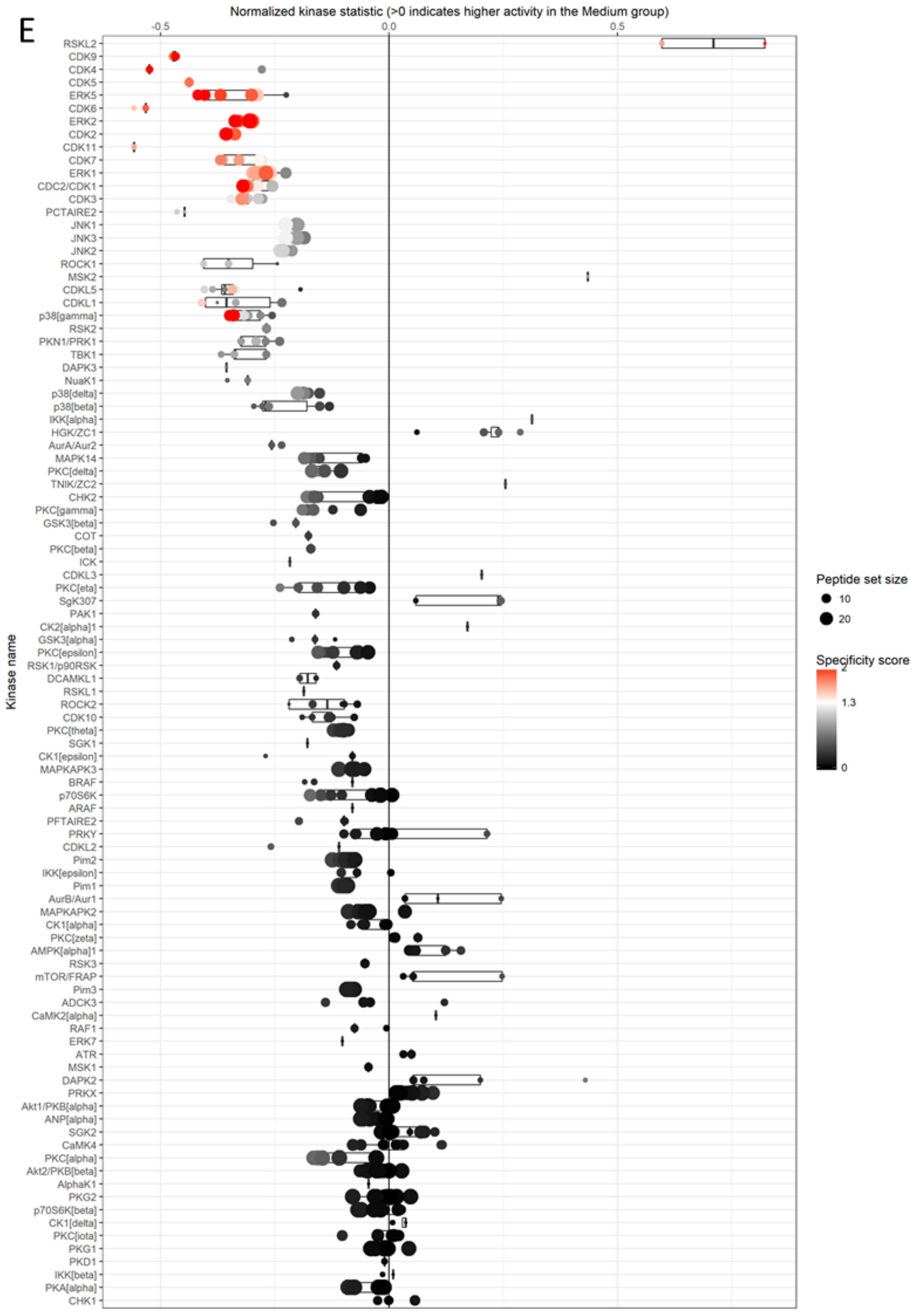
E
Normalized kinase statistic (>0 indicates higher activity in the Medium group)
-0.5
0.0
0.5
Kinase name
RSKL2
CDK9
CDK4
CDK5
ERK5
CDK6
ERK2
CDK2
CDK11
CDK7
ERK1
CDC2/CDK1
CDK3
PCTAIRE2
JNK1
JNK3
JNK2
ROCK1
MSK2
CDKL5
CDKL1
p38[gamma]
RSK2
PKN1/PRK1
TBK1
DAPK3
NuaK1
p38[delta]
p38[beta]
IKK[alpha]
HGK/ZC1
AurA/Aur2
MAPK14
PKC[delta]
TNIK/ZC2
CHK2
PKC[gamma]
GSK3[beta]
COT
PKC[beta]
ICK
CDKL3
PKC[eta]
SgK307
PAK1
CK2[alpha]1
GSK3[alpha]
PKC[epsilon]
RSK1/p90RSK
DCAMKL1
RSKL1
ROCK2
CDK10
PKC[theta]
SGK1
CK1[epsilon]
MAPKAPK3
BRAF
p70S6K
ARAF
PFTAIRE2
PRKY
CDKL2
Pim2
IKK[epsilon]
Pim1
AurB/Aur1
MAPKAPK2
CK1[alpha]
PKC[zeta]
AMPK[alpha]1
RSK3
mTOR/FRAP
Pim3
ADCK3
CaMK2[alpha]
RAF1
ERK7
ATR
MSK1
DAPK2
PRKX
Akt1/PKB[alpha]
ANP[alpha]
SGK2
CaMK4
PKC[alpha]
Akt2/PKB[beta]
AlphaK1
PKG2
p70S6K[beta]
CK1[delta]
PKC[iota]
PKG1
PKD1
IKK[beta]
PKA[alpha]
CHK1
Peptide set size
10
20
Specificity score
2
1.3
0

F

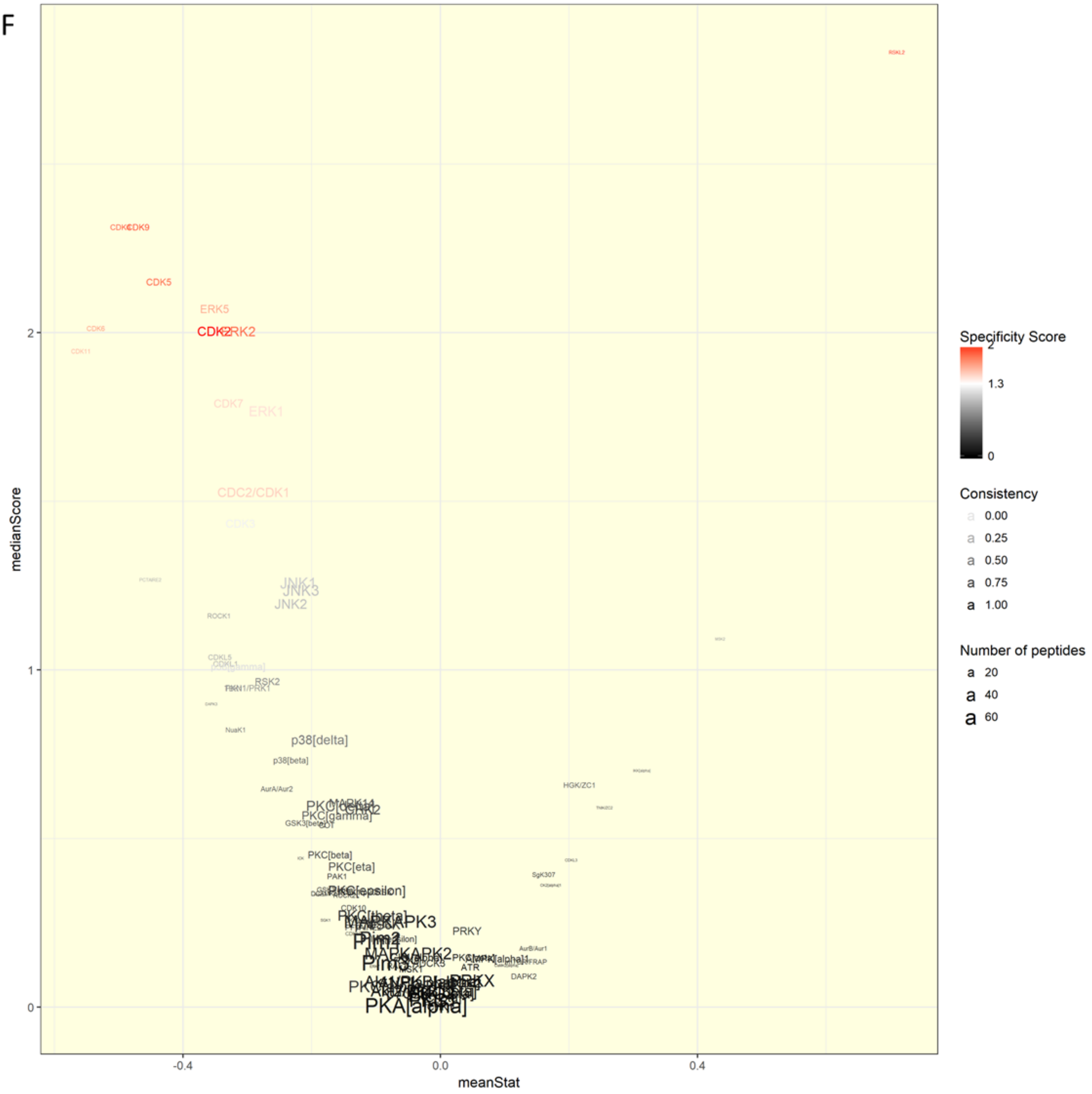
medianScore
meanStat
-0.4
0.0
0.4
0
1
2
Specificity Score
2
1.3
0
Consistency
0.00
0.25
0.50
0.75
1.00
Number of peptides
20
40
60
CDK9
CDK5
ERK5
ERK2
CDK7
ERK1
CDC2/CDK1
JNK1
JNK3
JNK2
ROCK1
RSK2
NuaK1
p38[delta]
p38[beta]
AurA/Aur2
HGK/ZC1
PKC[gamma]
PKC[beta]
PKC[eta]
PAK1
SgK307
PRKY
ATR
AurB/Aur1
DAPK2
PKA[alpha]

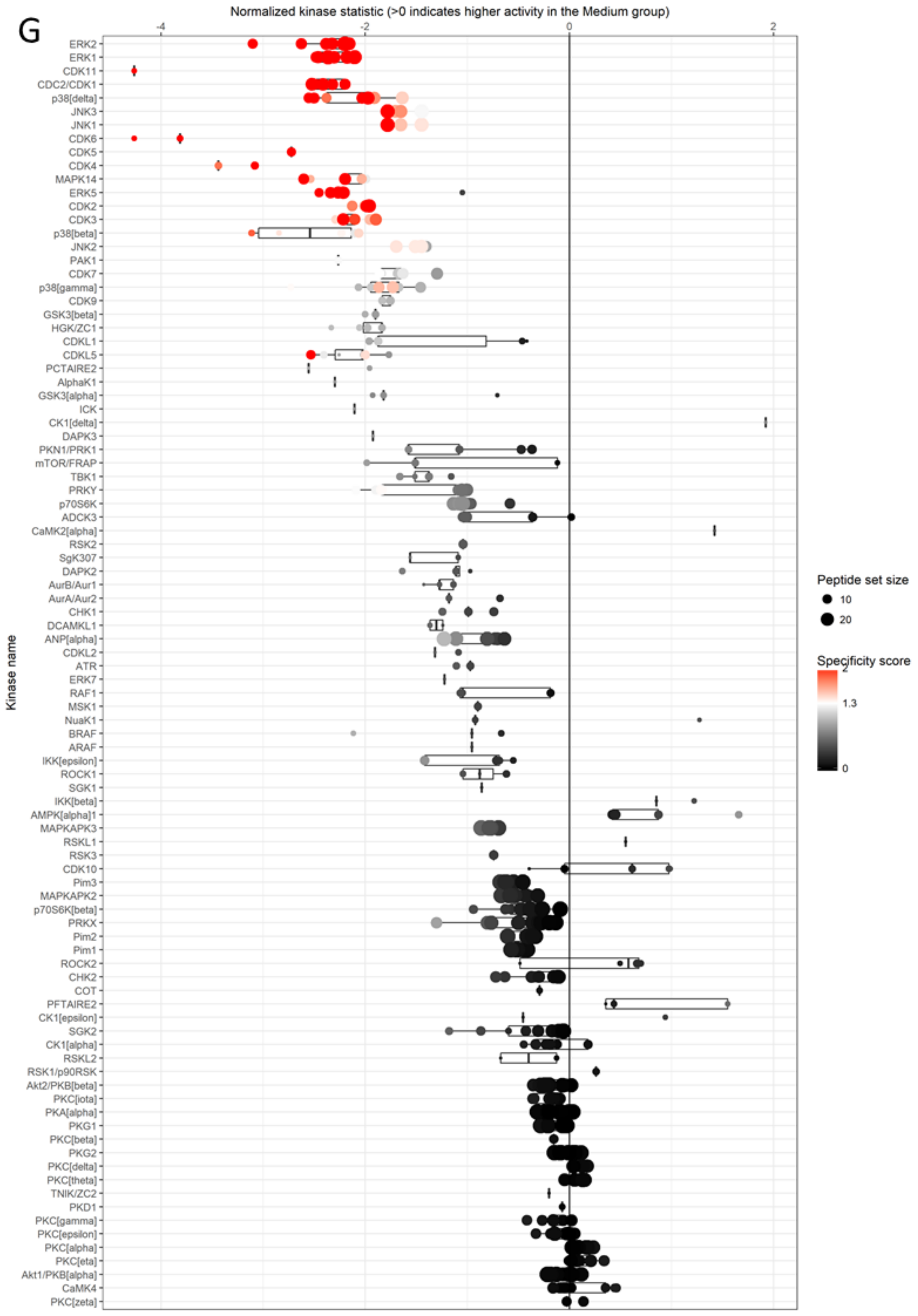
G
Normalized kinase statistic (>0 indicates higher activity in the Medium group)
-4
-2
0
2
Kinase name
ERK2
ERK1
CDK11
CDC2/CDK1
p38[delta]
JNK3
JNK1
CDK6
CDK5
CDK4
MAPK14
ERK5
CDK2
CDK3
p38[beta]
JNK2
PAK1
CDK7
p38[gamma]
CDK9
GSK3[beta]
HGK/ZC1
CDKL1
CDKL5
PCTAIRE2
AlphaK1
GSK3[alpha]
ICK
CK1[delta]
DAPK3
PKN1/PRK1
mTOR/FRAP
TBK1
PRKY
p70S6K
ADCK3
CaMK2[alpha]
RSK2
SgK307
DAPK2
AurB/Aur1
AurA/Aur2
CHK1
DCAMKL1
ANP[alpha]
CDKL2
ATR
ERK7
RAF1
MSK1
NuaK1
BRAF
ARAF
IKK[epsilon]
ROCK1
SGK1
IKK[beta]
AMPK[alpha]1
MAPKAPK3
RSKL1
RSK3
CDK10
Pim3
MAPKAPK2
p70S6K[beta]
PRKX
Pim2
Pim1
ROCK2
CHK2
COT
PFTAIRE2
CK1[epsilon]
SGK2
CK1[alpha]
RSKL2
RSK1/p90RSK
Akt2/PKB[beta]
PKC[iota]
PKA[alpha]
PKG1
PKC[beta]
PKG2
PKC[delta]
PKC[theta]
TNIK/ZC2
PKD1
PKC[gamma]
PKC[epsilon]
PKC[alpha]
PKC[eta]
Akt1/PKB[alpha]
CaMK4
PKC[zeta]
Peptide set size
10
20
Specificity score
2
1.3
0

H

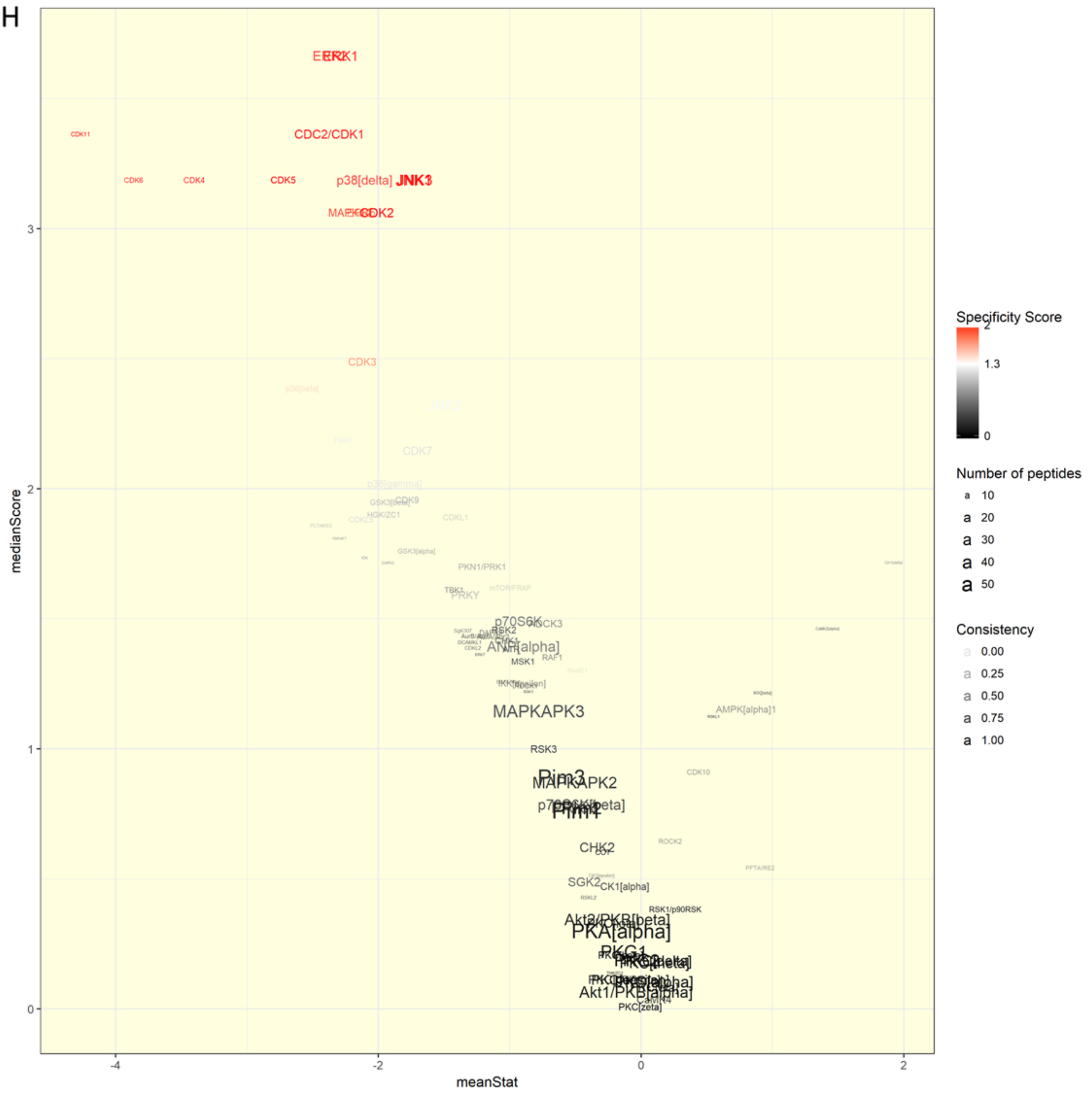

Suppl. Figure 8: Scoreplots and volcanoplots of STK upstream kinase analysis: A: Scoreplot of STK-Upstream kinase analysis for NCI-H2052 cells. B: Volcanoplot of STK-Upstream kinase analysis for NCI-H2052 cells. C: Scoreplot of STK-Upstream kinase analysis for NCI-H2452 cells. D: Volcanoplot of STK-Upstream kinase analysis for NCI-H2452 cells. E: Scoreplot of STK-Upstream kinase analysis for MSTO-211H cells. F: Volcanoplot of STK-Upstream kinase analysis for MSTO-211H cells. G: Scoreplot of STK-Upstream kinase analysis for MRC-5cells. H: Volcanoplot of STK-Upstream kinase analysis for MRC-5 cells.

A

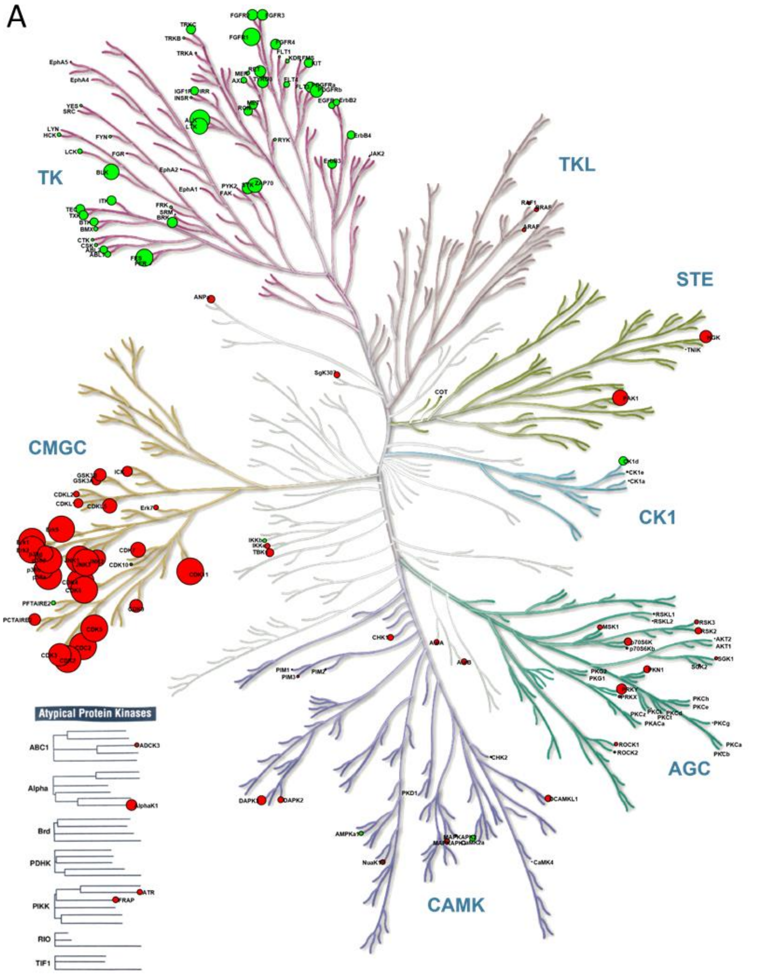

"Illustration reproduced courtesy of Cell Signaling Technology, Inc. (www.cellsignal.com)"

B

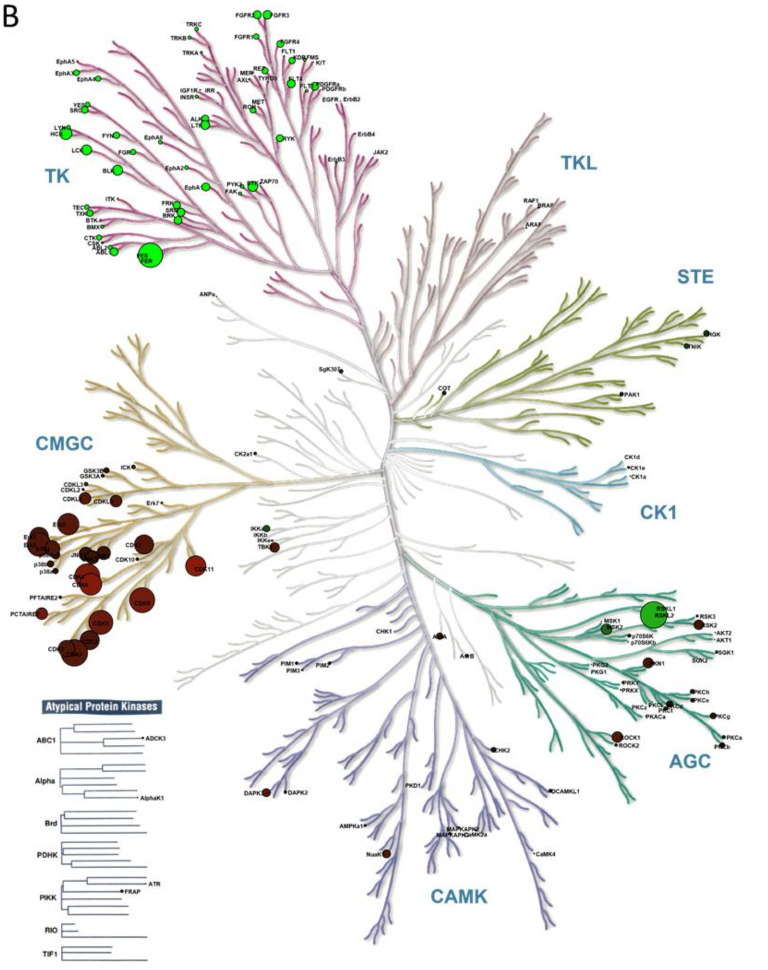

"Illustration reproduced courtesy of Cell Signaling Technology, Inc. (www.cellsignal.com)"

C

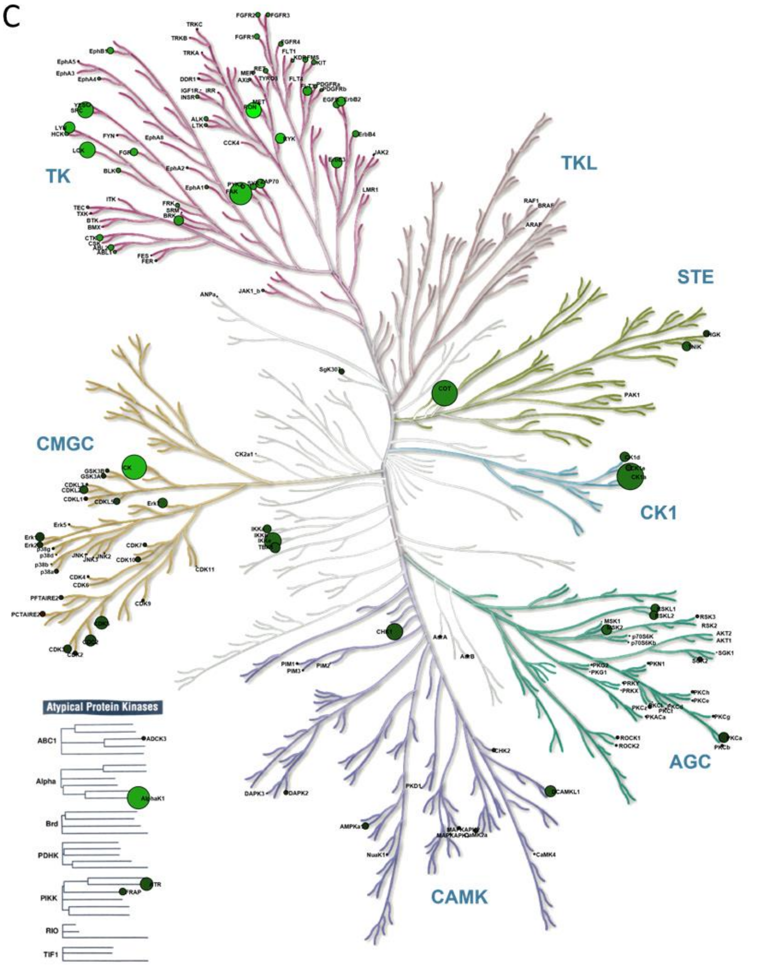

"Illustration reproduced courtesy of Cell Signaling Technology, Inc. (www.cellsignal.com)"

D

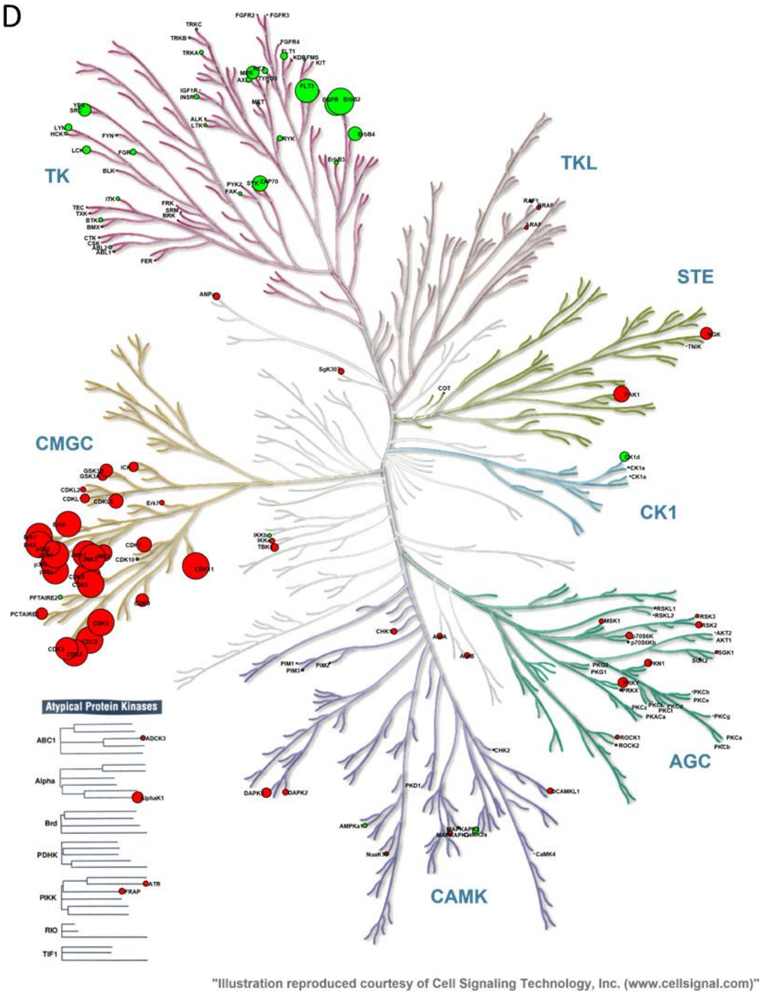

Suppl. Figure 9: Kinase-tree of A: NCI-H2052, B: MSTO-211H, C: NCI-H2452, D: MRC-5.

Suppl. Table 1: p-values of the influenced phosphosites during Cisplatin-therapy.

Phosphosite	**p-value**	**Estimate**
Protein phosphatase 1 regulatory subunit 1A (Protein phosphataseinhibitor 1) (IPP-1) (I-1)._PPR1A_28_40Q13522	0.000114062	0.42432336
Tyrosine-protein phosphatase non-receptor type 11 (EC 3.1.3.48)(Protein-tyrosine phosphatase 2C) (PTP-2C) (PTP-1D) (SH-PTP3) (SH-PTP2) (SHP-2) (Shp2)._PTN11_57_67_Q06124	0.000143306	-48.31918321
Proto-oncogene tyrosine-protein kinase receptor ret precursor(EC 2.7.10.1) (C-ret)._RET_1022_1034_P07949	0.0002297	-25.66823785
3-phosphoinositide-dependent protein kinase 1 (EC 2.7.11.1) (hPDK1)._PDPK1_369_381_O15530	0.000235799	-10.37735929
Proto-oncogene tyrosine-protein kinase FER (EC 2.7.10.2) (p94-FER) (c-FER) (Tyrosine kinase 3)._FER_707_719_P16591	0.000259019	-14.28773574
B- and T-lymphocyte attenuator,B- and T-lymphocyte-associated protein,CD272_BTLA_252_262_Q7Z6A9	0.000269051	-11.13993716
Gamma-enolase (EC 4.2.1.11) (2-phospho-D-glycerate hydro-lyase)(Neural enolase) (Neuron-specific enolase) (NSE) (Enolase 2)._ENOG_37_49_P09104	0.000396245	-125.8537697
Tyrosine-protein kinase ZAP-70 (EC 2.7.10.2) (70 kDa zeta-associatedprotein) (Syk-related tyrosine kinase)._ZAP70_313_325_P43403	0.000411389	-73.30975042
Beta-type platelet-derived growth factor receptor precursor(EC 2.7.10.1) (PDGF-R-beta) (CD140b antigen)._PGFRB_572_584_P09619	0.000488281	-31.33962259
3-phosphoinositide-dependent protein kinase 1 (EC 2.7.11.1) (hPDK1)._PDPK1_2_14_O15530	0.000530694	-15.57389841
Tyrosine-protein kinase JAK1 (EC 2.7.10.2) (Janus kinase 1) (JAK-1)._JAK1_ 1027_1039_P23458	0.000567748	-9.349056721
Mast/stem cell growth factor receptor Kit (EC:2.7.10.1), CD117_KIT_930_942_C942S_ P10721	0.000582714	-17.63207285
Insulin receptor substrate 1_IRS1_890_902_P35568	0.000697113	-6.5880502
Paxillin._PAXI_24_36_P49023	0.000770496	-23.34276715
Paxillin._PAXI_111_123_P49023	0.000918356	-46.3726396
Embryonal Fyn-associated substrate (HEFS)._EFS_246_258_O43281	0.001000346	-193.6509489
B-cell antigen receptor complex-associated protein alpha-chainprecursor (Ig-alpha) (MB-1 membrane glycoprotein) (Surface IgM-associated protein) (Membrane-bound immunoglobulin-associated protein)(CD79a antigen)._CD79A_181_193_P11912	0.00104713	-216.3446948
Hepatocyte growth factor receptor precursor (EC 2.7.10.1) (HGFreceptor) (Scatter factor receptor) (SF receptor) (HGF/SF receptor)(Met proto-oncogene tyrosine kinase) (c-Met)._MET_1227_1239_P08581	0.001292142	-12.23113142
RAC-alpha serine/threonine-protein kinase (EC:2.7.11.1)(PKB, RAC)_AKT1_320_332_P31749	0.001382244	-4.611634874
Paired mesoderm homeobox protein 2 (PRX-2) (Paired-related homeoboxprotein 2)._PRRX2_202_214_Q99811	0.001409165	-11.72484268
RAF proto-oncogene serine/threonine-protein kinase (EC 2.7.11.1) (Raf-1) (C-RAF) (cRaf)._RAF1_332_344_P04049	0.00156752	-11.69496949
Ephrin type-A receptor 7 precursor (EC 2.7.10.1) (Tyrosine-proteinkinase receptor EHK-3) (EPH homology kinase 3)	0.001646125	-14.60849123

(Receptor protein-tyrosine kinase HEK11)._EPHA7_607_619_Q15375		
Hepatitis A virus cellular receptor 2, T-cell immunoglobulin and mucin domain-containing protein 3,T-cell membrane protein 3_HAVR2_257_267_Q8TDQ0	0.001651309	-40.50157276
Vascular endothelial growth factor receptor 2 precursor (EC 2.7.10.1)(VEGFR-2) (Kinase insert domain receptor) (Protein-tyrosine kinasereceptor Flk-1) (CD309 antigen)._VGFR2_989_1001_P35968	0.00176521	-27.45754335
Ras GTPase-activating protein 1 (GTPase-activating protein) (GAP) (Rasp21 protein activator) (p120GAP) (RasGAP)._RASA1_453_465_P20936	0.001815013	-14.12264248
Phosphatidylinositol 3-kinase regulatory subunit alpha (PI3-kinase p85subunit alpha) (PtdIns-3-kinase p85-alpha) (PI3K)._P85A_600_612_P27986	0.00198713	-29.80188591
Proto-oncogene tyrosine-protein kinase Fes/Fps (EC 2.7.10.2) (C-Fes)._FES_706_718_P07332	0.002251143	-21.9072331
Tyrosine-protein phosphatase non-receptor type 6 (EC:3.1.3.48)_PTN6_558_570_P29350	0.002386595	-18.07075475
1-phosphatidylinositol 4,5-bisphosphate phosphodiesterase gamma-2 (EC:3.1.4.11) (Phosphoinositide phospholipase C-gamma-2) (PLC-IV) (Phospholipase C-gamma-2)(PLC-gamma-2)_PLCG2_1191_1203_C1200S_P16885	0.002475367	-61.20283304
Tyrosine-protein kinase FRK (EC 2.7.10.2) (FYN-related kinase)(Nuclear tyrosine protein kinase RAK)._FRK_380_392_P42685	0.002520094	-55.28459362
Protein 4.1 (Band 4.1) (P4.1) (EPB4.1) (4.1R)._41_654_666_P11171	0.00263458	-15.04559945
Cyclin-dependent kinase 1 (EC:2.7.11.22, EC:2.7.11.23), Cell division protein kinase 1, Cell division control protein 2 homolog, (p34 protein kinase)(CDK1)_CDK1_9_21_P06493	0.002646862	-26.94654036
Glycogen synthase kinase-3 beta (EC:2.7.11.26), Serine/threonine-protein kinase GSK3B (EC:2.7.11.1)_GSK3B_210_222_C218S_P49841	0.00267152	-7.509434261
Platelet endothelial cell adhesion molecule precursor (PECAM-1)(EndoCAM) (GPIIA') (CD31 antigen)._PECA1_708_718_P16284	0.002672271	-74.29088238
Tyrosine-protein phosphatase non-receptor type 6 (EC:3.1.3.48)_PTN6_531_541_P29350	0.002785303	-72.1194981
Serine/threonine-protein phosphatase 2A catalytic subunit beta isoform(EC 3.1.3.16) (PP2A-beta)._PP2AB_297_309_P62714	0.003305101	-5.710691829
Forkhead box protein O3 (Forkhead in rhabdomyosarcoma-like 1) (AF6q21protein)._FOXO3_25_37O43524	0.003417969	0.293575883
T-cell surface glycoprotein CD3 epsilon chain, T-cell surface antigen T3/Leu-4 epsilon chain, CD3e_CD3E_182_194_P07766	0.003457397	-15.84434021
Myelin protein zero-like protein 1_MPZL1_236_246_O95297	0.003637426	-16.03302167
Cyclin-dependent kinase 2 (EC:2.7.11.22) Cell division protein kinase 2 (EC 2.7.11.22) (p33 protein kinase)._CDK2_8_20_P24941	0.004066679	-38.38521916
1-phosphatidylinositol-4,5-bisphosphate phosphodiesterase gamma-1(EC 3.1.4.11) (Phosphoinositide phospholipase C) (PLC-gamma-1)(Phospholipase C-gamma-1) (PLC-II) (PLC-148)._PLCG1_764_776_P19174	0.004119601	-80.45754493
Platelet endothelial cell adhesion molecule precursor (PECAM-1)(EndoCAM) (GPIIA') (CD31	0.004132181	-26.66509413

antigen)._PECA1_706_718_P16284		
Insulin receptor substrate 2_IRS2_626_638_Q9Y4H2	0.00418241	-22.23899372
NA_ART_004_EAIYAAPFAKKKXC_NA	0.004183845	-111.8191821
Ephrin type-A receptor 1 precursor (EC 2.7.10.1) (Tyrosine-proteinkinase receptor EPH)._EPHA1_774_786_P21709	0.004781317	-17.04559706
Protein tyrosine kinase 2 beta (EC 2.7.10.2) (Focal adhesion kinase 2)(FADK 2) (Proline-rich tyrosine kinase 2) (Cell adhesion kinase beta)(CAK beta) (Calcium-dependent tyrosine kinase) (CADTK) (Relatedadhesion focal tyrosine kinase) (RAFTK)._FAK2_572_584_Q14289	0.005257955	-13.58176041
Tyrosine-protein kinase JAK2 (EC 2.7.10.2) (Janus kinase 2) (JAK-2)._JAK2_563_577_O60674	0.005453912	-8.149371153
Hepatocyte growth factor receptor precursor (EC 2.7.10.1) (HGFreceptor) (Scatter factor receptor) (SF receptor) (HGF/SF receptor)(Met proto-oncogene tyrosine kinase) (c-Met)._MET_1228_1240_P08581	0.00722373	-6.194968485
Ephrin type-A receptor 2 precursor (EC 2.7.10.1) (Tyrosine-proteinkinase receptor ECK) (Epithelial cell kinase)._EPHA2_765_777_P29317	0.007626438	-17.61792463
Proto-oncogene tyrosine-protein kinase LCK (EC 2.7.10.2) (p56-LCK)(Lymphocyte cell-specific protein-tyrosine kinase) (LSK) (T cell-specific protein-tyrosine kinase)._LCK_387_399_P06239	0.008333806	-8.970125555
Receptor tyrosine-protein kinase erbB-2 precursor (EC 2.7.10.1)(p185erbB2) (C-erbB-2) (NEU proto-oncogene) (Tyrosine kinase-type cellsurface receptor HER2) (MLN 19) (CD340 antigen)._ERBB2_870_882_P04626	0.008530882	-6.504717132
Tyrosine-protein phosphatase non-receptor type 12 (EC 3.1.3.48)(Protein-tyrosine phosphatase G1) (PTPG1) (PTP-PEST)._PTN12_32_44Q05209	0.009277344	-0.451752543
Linker for activation of T-cells family member 1 (36 kDa phospho-tyrosine adapter protein) (pp36) (p36-38)._LAT_249_261_O43561	0.009424028	-10.78773577
Mitogen-activated protein kinase 12 (EC 2.7.11.24) (Extracellularsignal-regulated kinase 6) (ERK-6) (ERK5) (Stress-activated proteinkinase 3) (Mitogen-activated protein kinase p38 gamma) (MAP kinase p38gamma)._MK12_180_189_M182B_P53778	0.009425328	-5.045597474

Suppl. Table 2: Significantly altered phosphorylation levels between the different cell lines.

Phosphosite	**Cell lines**	**p-value**	**Estimate**
NA_ART_004_EAIYAAPFAKKKXC_NA	MRC5 by H2052	0.002558007	-239.5974795
Epidermal growth factor receptor precursor (EC 2.7.10.1) (Receptortyrosine-protein kinase ErbB-1)._EGFR_1165_1177_P00533	MRC5 by H2052	0.005451835	-14.9308176
Focal adhesion kinase 1 (EC 2.7.10.2) (FADK 1) (pp125FAK) (Protein-tyrosine kinase 2)._FAK1_569_581_Q05397	MRC5 by H2052	0.000187356	-18.30188672
Tyrosine-protein kinase JAK2 (EC 2.7.10.2) (Janus kinase 2) (JAK-2)._JAK2_563_577_O60674	MRC5 by H2052	0.005468379	-32.40251571

Hepatocyte growth factor receptor precursor (EC 2.7.10.1) (HGFreceptor) (Scatter factor receptor) (SF receptor) (HGF/SF receptor)(Met proto-oncogene tyrosine kinase) (c-Met)._MET_1227_1239_P08581	MRC5 by H2052	0.003539214	-39.30817453
Hepatocyte growth factor receptor precursor (EC 2.7.10.1) (HGFreceptor) (Scatter factor receptor) (SF receptor) (HGF/SF receptor)(Met proto-oncogene tyrosine kinase) (c-Met)._MET_1228_1240_P08581	MRC5 by H2052	0.001672374	-25.22641466
3-phosphoinositide-dependent protein kinase 1 (EC 2.7.11.1) (hPDK1)._PDPK1_2_14_O15530	MRC5 by H2052	0.008324809	-53.77987242
Paired mesoderm homeobox protein 2 (PRX-2) (Paired-related homeoboxprotein 2)._PRRX2_202_214_Q99811	MRC5 by H2052	0.002741309	-28.35220162
Tyrosine-protein phosphatase non-receptor type 6 (EC:3.1.3.48)_PTN6_558_570_P29350	MRC5 by H2052	0.004389	-82.03773563
RAF proto-oncogene serine/threonine-protein kinase (EC 2.7.11.1) (Raf-1) (C-RAF) (cRaf)._RAF1_332_344_P04049	MRC5 by H2052	0.001429345	-37.70440292
Tyrosine-protein kinase ZAP-70 (EC 2.7.10.2) (70 kDa zeta-associatedprotein) (Syk-related tyrosine kinase)._ZAP70_313_325_P43403	MRC5 by H2052	0.008248634	-289.4213893
B-cell antigen receptor complex-associated protein alpha-chainprecursor (Ig-alpha) (MB-1 membrane glycoprotein) (Surface IgM-associated protein) (Membrane-bound immunoglobulin-associated protein)(CD79a antigen)._CD79A_181_193_P11912	MRC5 by H2452	0.008002374	-973.6477613
Embryonal Fyn-associated substrate (HEFS)._EFS_246_258_O43281	MRC5 by H2452	0.001113984	-761.9623006
Gamma-enolase (EC 4.2.1.11) (2-phospho-D-glycerate hydro-lyase)(Neural enolase) (Neuron-specific enolase) (NSE) (Enolase 2)._ENOG_37_49_P09104	MRC5 by H2452	0.000322357	-509.7043889
Ephrin type-A receptor 7 precursor (EC 2.7.10.1) (Tyrosine-proteinkinase receptor EHK-3) (EPH homology kinase 3) (Receptor protein-tyrosine kinase HEK11)._EPHA7_607_619_Q15375	MRC5 by H2452	0.001788365	-44.49056577
Protein tyrosine kinase 2 beta (EC 2.7.10.2) (Focal adhesion kinase 2)(FADK 2) (Proline-rich tyrosine kinase 2) (Cell adhesion kinase beta)(CAK beta) (Calcium-dependent tyrosine kinase) (CADTK) (Relatedadhesion focal tyrosine kinase) (RAFTK)._FAK2_572_584_Q14289	MRC5 by H2452	0.004303637	-40.8742129
Proto-oncogene tyrosine-protein kinase Fes/Fps (EC 2.7.10.2) (C-Fes)._FES_706_718_P07332	MRC5 by H2452	0.00545785	-84.94339617
Tyrosine-protein kinase FRK (EC 2.7.10.2) (FYN-related kinase)(Nuclear tyrosine protein kinase RAK)._FRK_380_392_P42685	MRC5 by H2452	0.004686346	-211.4654141
Insulin receptor substrate 1_IRS1_890_902_P35568	MRC5 by H2452	0.006774102	-14.11320752
Tyrosine-protein kinase JAK3 (EC:2.7.10.2)	MRC5 by	0.009318529	-17.84905609

(Janus kinase 3) (JAK-3)._JAK3_974_986_P52333	H2452		
Mast/stem cell growth factor receptor Kit (EC:2.7.10.1), CD117_KIT_930_942_C942S_ P10721	MRC5 by H2452	0.009808788	-50.81133427
Phosphatidylinositol 3-kinase regulatory subunit alpha (PI3-kinase p85subunit alpha) (PtdIns-3-kinase p85-alpha) (PI3K)._P85A_600_612_P27986	MRC5 by H2452	0.007955423	-85.52829837
Paxillin._PAXI_24_36_P49023	MRC5 by H2452	0.006774193	-73.28930728
1-phosphatidylinositol-4,5-bisphosphate phosphodiesterase gamma-1(EC 3.1.4.11) (Phosphoinositide phospholipase C) (PLC-gamma-1)(Phospholipase C-gamma-1) (PLC-II) (PLC-148)._PLCG1_764_776_P19174	MRC5 by H2452	0.006186425	-244.6666616
Tyrosine-protein phosphatase non-receptor type 11 (EC 3.1.3.48)(Protein-tyrosine phosphatase 2C) (PTP-2C) (PTP-1D) (SH-PTP3) (SH-PTP2) (SHP-2) (Shp2)._PTN11_57_67_Q06124	MRC5 by H2452	0.001872277	-156.1194992
Tyrosine-protein phosphatase non-receptor type 6 (EC:3.1.3.48)_PTN6_531_541_P29350	MRC5 by H2452	0.000818014	-271.3144646
T-cell surface glycoprotein CD3 zeta chain precursor (T-cell receptorT3 zeta chain) (CD247 antigen)._CD3Z_77_89_P20963	MSTO211H by H2452	0.002727064	7.94339625
Embryonal Fyn-associated substrate (HEFS)._EFS_246_258_O43281	MSTO211H by H2452	0.009897944	-432.8365071
Epidermal growth factor receptor precursor (EC 2.7.10.1) (Receptortyrosine-protein kinase ErbB-1)._EGFR_1165_1177_P00533	MSTO211H by H2452	0.000151912	12.24528249
Gamma-enolase (EC 4.2.1.11) (2-phospho-D-glycerate hydro-lyase)(Neural enolase) (Neuron-specific enolase) (NSE) (Enolase 2)._ENOG_37_49_P09104	MSTO211H by H2452	0.004879687	-304.5974783
Ephrin type-A receptor 7 precursor (EC 2.7.10.1) (Tyrosine-proteinkinase receptor EHK-3) (EPH homology kinase 3) (Receptor protein-tyrosine kinase HEK11)._EPHA7_607_619_Q15375	MSTO211H by H2452	0.009854728	-8.18867747
Glycogen synthase kinase-3 beta (EC:2.7.11.26), Serine/threonine-protein kinase GSK3B (EC:2.7.11.1)_GSK3B_210_222_C218S_P49841	MSTO211H by H2452	0.006404667	11.28930664
Tyrosine-protein phosphatase non-receptor type 6 (EC:3.1.3.48)_PTN6_531_541_P29350	MSTO211H by H2452	0.001284386	-211.698115
40S ribosomal protein S6 (Phosphoprotein NP33)._RS6_228_240P62753	MSTO211H by MRC5	0.007080003	1.095669269
T-cell surface glycoprotein CD3 zeta chain precursor (T-cell receptorT3 zeta chain) (CD247 antigen)._CD3Z_77_89_P20963	MSTO211H by MRC5	0.00769951	14.56603774
B-cell antigen receptor complex-associated protein alpha-chainprecursor (Ig-alpha) (MB-1 membrane glycoprotein) (Surface IgM-associated protein) (Membrane-bound immunoglobulin-associated protein)(CD79a	MSTO211H by MRC5	0.003516668	379.2829946

antigen)._CD79A_181_193_P11912			
Cyclin-dependent kinase 1 (EC:2.7.11.22, EC:2.7.11.23), Cell division protein kinase 1, Cell division control protein 2 homolog, (p34 protein kinase)(CDK1)_CDK1_9_21_P06493	MSTO211H by MRC5	0.007146253	30.58490436
Embryonal Fyn-associated substrate (HEFS)._EFS_246_258_O43281	MSTO211H by MRC5	0.00471398	329.1257935
Ephrin type-A receptor 2 precursor (EC 2.7.10.1) (Tyrosine-proteinkinase receptor ECK) (Epithelial cell kinase)._EPHA2_765_777_P29317	MSTO211H by MRC5	0.002668944	25.33333428
Ephrin type-A receptor 7 precursor (EC 2.7.10.1) (Tyrosine-proteinkinase receptor EHK-3) (EPH homology kinase 3) (Receptor protein-tyrosine kinase HEK11)._EPHA7_607_619_Q15375	MSTO211H by MRC5	0.004014922	36.3018883
Erythropoietin receptor precursor (EPO-R)._EPOR_361_373_P19235	MSTO211H by MRC5	0.008806221	21.46540902
Protein tyrosine kinase 2 beta (EC 2.7.10.2) (Focal adhesion kinase 2)(FADK 2) (Proline-rich tyrosine kinase 2) (Cell adhesion kinase beta)(CAK beta) (Calcium-dependent tyrosine kinase) (CADTK) (Relatedadhesion focal tyrosine kinase) (RAFTK)._FAK2_572_584_Q14289	MSTO211H by MRC5	0.000321329	42.30817668
Proto-oncogene tyrosine-protein kinase Fes/Fps (EC 2.7.10.2) (C-Fes)._FES_706_718_P07332	MSTO211H by MRC5	0.000607493	39.66666786
Tyrosine-protein kinase FRK (EC 2.7.10.2) (FYN-related kinase)(Nuclear tyrosine protein kinase RAK)._FRK_380_392_P42685	MSTO211H by MRC5	0.005351618	60.75471815
Hepatitis A virus cellular receptor 2, T-cell immunoglobulin and mucin domain-containing protein 3,T-cell membrane protein 3_HAVR2_257_267_Q8TDQ0	MSTO211H by MRC5	0.000299596	43.13207626
Insulin receptor substrate 2_IRS2_626_638_Q9Y4H2	MSTO211H by MRC5	0.005628554	51.3584919
Tyrosine-protein kinase JAK1 (EC 2.7.10.2) (Janus kinase 1) (JAK-1)._JAK1_ 1027_1039_P23458	MSTO211H by MRC5	0.002975482	13.295597
Tyrosine-protein kinase JAK3 (EC:2.7.10.2) (Janus kinase 3) (JAK-3)._JAK3_974_986_P52333	MSTO211H by MRC5	0.002721384	17.81761026
Mast/stem cell growth factor receptor Kit (EC:2.7.10.1), CD117_KIT_930_942_C942S_ P10721	MSTO211H by MRC5	0.008217992	30.7044153
Hepatocyte growth factor receptor precursor (EC 2.7.10.1) (HGFreceptor) (Scatter factor receptor) (SF receptor) (HGF/SF receptor)(Met proto-oncogene tyrosine kinase) (c-Met)._MET_1227_1239_P08581	MSTO211H by MRC5	0.007490657	32.71069122
Phosphatidylinositol 3-kinase regulatory subunit alpha (PI3-kinase p85subunit alpha) (PtdIns-3-kinase p85-alpha) (PI3K)._P85A_600_612_P27986	MSTO211H by MRC5	0.002986294	46.25157261

Paxillin._PAXI_111_123_P49023	MSTO211H by MRC5	0.005318965	78.83018685
Paxillin._PAXI_24_36_P49023	MSTO211H by MRC5	0.005119719	35.79245186
3-phosphoinositide-dependent protein kinase 1 (EC 2.7.11.1) (hPDK1)._PDPK1_2_14_O15530	MSTO211H by MRC5	0.008123918	42.51572243
Platelet endothelial cell adhesion molecule precursor (PECAM-1)(EndoCAM) (GPIIA') (CD31 antigen)._PECA1_706_718_P16284	MSTO211H by MRC5	0.004591573	46.02515896
Platelet endothelial cell adhesion molecule precursor (PECAM-1)(EndoCAM) (GPIIA') (CD31 antigen)._PECA1_708_718_P16284	MSTO211H by MRC5	0.007099719	103.9433982
1-phosphatidylinositol-4,5-bisphosphate phosphodiesterase gamma-1(EC 3.1.4.11) (Phosphoinositide phospholipase C) (PLC-gamma-1)(Phospholipase C-gamma-1) (PLC-II) (PLC-148)._PLCG1_764_776_P19174	MSTO211H by MRC5	0.002089885	114.2452901
Tyrosine-protein phosphatase non-receptor type 11 (EC 3.1.3.48)(Protein-tyrosine phosphatase 2C) (PTP-2C) (PTP-1D) (SH-PTP3) (SH-PTP2) (SHP-2) (Shp2)._PTN11_57_67_Q06124	MSTO211H by MRC5	0.005131532	81.39622497
Tyrosine-protein phosphatase non-receptor type 6 (EC:3.1.3.48)_PTN6_531_541_P29350	MSTO211H by MRC5	0.000436347	59.61634955
Proto-oncogene tyrosine-protein kinase receptor ret precursor(EC 2.7.10.1) (C-ret)._RET_1022_1034_P07949	MSTO211H by MRC5	0.000984409	48.71069209
Tyrosine-protein kinase Tec (EC 2.7.10.2)._TEC_512_524_P42680	MSTO211H by MRC5	0.009068564	15.28930807
Tyrosine-protein kinase ZAP-70 (EC 2.7.10.2) (70 kDa zeta-associatedprotein) (Syk-related tyrosine kinase)._ZAP70_485_497_P43403	MSTO211H by MRC5	0.008433828	17.47169737

Suppl. Table 3: Association of phosphosite phosphorylation ("+"= high, "-"= low phosphorylation) to apoptosis ratio of cells after cisplatin treatment.

Phosphosites	**Apoptosis grouping**	**Frequency**
CDK1_Y9_Y21 (CDK1) -	high apoptosis ratio	5
CDK1_Y9_Y21 (CDK1) -	low apoptosis ratio	1
CDK1_Y9_Y21 (CDK1) +	high apoptosis ratio	1
CDK1_Y9_Y21 (CDK1) +	low apoptosis ratio	5
CDK2_Y8_Y20 (CDK2) -	high apoptosis ratio	4
CDK2_Y8_Y20 (CDK2) -	low apoptosis ratio	2
CDK2_Y8_Y20 (CDK2) +	high apoptosis ratio	2
CDK2_Y8_Y20 (CDK2) +	low apoptosis ratio	4
CREB1_Y126(CREB1) -	high apoptosis ratio	5
CREB1_Y126(CREB1) -	low apoptosis ratio	1
CREB1_Y126(CREB1) +	high apoptosis ratio	1
CREB1_Y126(CREB1) +	low apoptosis ratio	5
EPHA2_Y765_Y777 (EPHA2) -	high apoptosis ratio	4
EPHA2_Y765_Y777 (EPHA2) -	low apoptosis ratio	2

EPHA2_Y765_Y777 (EPHA2) +	high apoptosis ratio	2
EPHA2_Y765_Y777 (EPHA2) +	low apoptosis ratio	4
ESR1_Y160 (ESR1) -	high apoptosis ratio	6
ESR1_Y160 (ESR1) -	low apoptosis ratio	0
ESR1_Y160 (ESR1) +	high apoptosis ratio	0
ESR1_Y160 (ESR1) +	low apoptosis ratio	6
FES_Y706_Y718 (FES) -	high apoptosis ratio	4
FES_Y706_Y718 (FES) -	low apoptosis ratio	2
FES_Y706_Y718 (FES) +	high apoptosis ratio	2
FES_Y706_Y718 (FES) +	low apoptosis ratio	4
GBRB2 _Y427 (GABRB2) -	high apoptosis ratio	5
GBRB2 _Y427 (GABRB2) -	low apoptosis ratio	1
GBRB2 _Y427 (GABRB2) +	high apoptosis ratio	1
GBRB2 _Y427 (GABRB2) +	low apoptosis ratio	5
HAVR2_Y257_Y267 (HAVCR2) -	high apoptosis ratio	5
HAVR2_Y257_Y267 (HAVCR2) -	low apoptosis ratio	1
HAVR2_Y257_Y267 (HAVCR2) +	high apoptosis ratio	1
HAVR2_Y257_Y267 (HAVCR2) +	low apoptosis ratio	5
KIT_Y930_Y942_C942S (KIT) -	high apoptosis ratio	5
KIT_Y930_Y942_C942S (KIT) -	low apoptosis ratio	1
KIT_Y930_Y942_C942S (KIT) +	high apoptosis ratio	1
KIT_Y930_Y942_C942S (KIT) +	low apoptosis ratio	5
LAT_Y249_Y261 (LAT) -	high apoptosis ratio	6
LAT_Y249_Y261 (LAT) -	low apoptosis ratio	0
LAT_Y249_Y261 (LAT) +	high apoptosis ratio	0
LAT_Y249_Y261 (LAT) +	low apoptosis ratio	6
P85A_Y600_Y612 (PIK3R1) -	high apoptosis ratio	5
P85A_Y600_Y612 (PIK3R1) -	low apoptosis ratio	1
P85A_Y600_Y612 (PIK3R1) +	high apoptosis ratio	1
P85A_Y600_Y612 (PIK3R1) +	low apoptosis ratio	5
PAXI_Y111_Y123 (PXN) -	high apoptosis ratio	5
PAXI_Y111_Y123 (PXN) -	low apoptosis ratio	1
PAXI_Y111_Y123 (PXN) +	high apoptosis ratio	1
PAXI_Y111_Y123 (PXN) +	low apoptosis ratio	5
PAXI_Y24_Y36 (PXN) -	high apoptosis ratio	4
PAXI_Y24_Y36 (PXN) -	low apoptosis ratio	2
PAXI_Y24_Y36 (PXN) +	high apoptosis ratio	2
PAXI_Y24_Y36 (PXN) +	low apoptosis ratio	4
PDPK1_Y2_Y14 (PDPK1) -	high apoptosis ratio	4
PDPK1_Y2_Y14 (PDPK1) -	low apoptosis ratio	2
PDPK1_Y2_Y14 (PDPK1) +	high apoptosis ratio	2
PDPK1_Y2_Y14 (PDPK1) +	low apoptosis ratio	4
PECA1_Y706_Y718 (PECAM1) -	high apoptosis ratio	4
PECA1_Y706_Y718 (PECAM1) -	low apoptosis ratio	2
PECA1_Y706_Y718 (PECAM1) +	high apoptosis ratio	2
PECA1_Y706_Y718 (PECAM1) +	low apoptosis ratio	4
PECA1_Y708_Y718 (PECAM1) -	high apoptosis ratio	5

PECA1_Y708_Y718 (PECAM1) -	low apoptosis ratio	1
PECA1_Y708_Y718 (PECAM1) +	high apoptosis ratio	1
PECA1_Y708_Y718 (PECAM1) +	low apoptosis ratio	5
PGFRB_Y572_Y584 (PDGFRB) -	high apoptosis ratio	5
PGFRB_Y572_Y584 (PDGFRB) -	low apoptosis ratio	1
PGFRB_Y572_Y584 (PDGFRB) +	high apoptosis ratio	1
PGFRB_Y572_Y584 (PDGFRB) +	low apoptosis ratio	5
PLCG2_Y1191_Y1203_C1200S (PLCG2) -	high apoptosis ratio	5
PLCG2_Y1191_Y1203_C1200S (PLCG2) -	low apoptosis ratio	1
PLCG2_Y1191_Y1203_C1200S (PLCG2) +	high apoptosis ratio	1
PLCG2_Y1191_Y1203_C1200S (PLCG2) +	low apoptosis ratio	5
PTN11_Y57_Y67 (PTPN11) -	high apoptosis ratio	4
PTN11_Y57_Y67 (PTPN11) -	low apoptosis ratio	2
PTN11_Y57_Y67 (PTPN11) +	high apoptosis ratio	2
PTN11_Y57_Y67 (PTPN11) +	low apoptosis ratio	4
PTN12_Y32 (PTPN12) -	high apoptosis ratio	6
PTN12_Y32 (PTPN12) -	low apoptosis ratio	0
PTN12_Y32 (PTPN12) +	high apoptosis ratio	0
PTN12_Y32 (PTPN12) +	low apoptosis ratio	6
PTN6_Y531_Y541 (PTPN6) -	high apoptosis ratio	5
PTN6_Y531_Y541 (PTPN6) -	low apoptosis ratio	1
PTN6_Y531_Y541 (PTPN6) +	high apoptosis ratio	1
PTN6_Y531_Y541 (PTPN6) +	low apoptosis ratio	5
PTN6_Y558_Y570 (PTPN6) -	high apoptosis ratio	6
PTN6_Y558_Y570 (PTPN6) -	low apoptosis ratio	0
PTN6_Y558_Y570 (PTPN6) +	high apoptosis ratio	0
PTN6_Y558_Y570 (PTPN6) +	low apoptosis ratio	6
RASA1_Y453_Y465 (RASA1) -	high apoptosis ratio	5
RASA1_Y453_Y465 (RASA1) -	low apoptosis ratio	1
RASA1_Y453_Y465 (RASA1) +	high apoptosis ratio	1
RASA1_Y453_Y465 (RASA1) +	low apoptosis ratio	5
RS6_Y228 (RPS6) -	high apoptosis ratio	5
RS6_Y228 (RPS6) -	low apoptosis ratio	1
RS6_Y228 (RPS6) +	high apoptosis ratio	1
RS6_Y228 (RPS6) +	low apoptosis ratio	5
